# Analysis of the Expression and Role of Keratin 17 in Human Tumors

**DOI:** 10.3389/fgene.2022.801698

**Published:** 2022-05-12

**Authors:** Hanqun Zhang, Yun Zhang, Zhiyu Feng, Liang Lu, Yong Li, Yuncong Liu, Yanping Chen

**Affiliations:** ^1^ Department of Oncology, Guizhou Provincial People’s Hospital, Guiyang, China; ^2^ Department of Pathology, Guizhou Provincial People’s Hospital, Guiyang, China

**Keywords:** Krt17, carcinogenesis, mechanism, prognosis, cancer

## Abstract

**Objective:** We aimed to explore the expression and carcinogenic effect of KRT17 in human tumors and provide useful information for the study of KRT17.

**Methods:** We used databases including the Cancer Genome Atlas, Gene Expression Omnibus, GTEx, and GEPIA2 to analyze the expression, mutation, and prognosis of KRT17 in human tumors. Through webservers, including UALCAN, TIMER2.0, and STRING, we learned about the genetic variation, immune cell penetration, and enrichment analysis of KRT17-related genes.

**Results:** KRT17 was highly expressed in most tumors (such as esophageal cancer, lung cancer, cervical cancer, etc.), and the high expression level correlated with tumor stage and prognosis. In addition, amplification was the main type of KRT17 tumor variation, with an amplification rate of about 9%, followed by mutation, with a mutation rate of 4%. Moreover, KRT17 was strongly associated with tumor-infiltrating immune cells (such as macrophages, CD8+T, Tregs, and cancer-associated fibroblasts). KEGG analysis suggested that KRT17 may play a role in tumor pathogenesis following human papillomavirus infection, and the gene ontology enrichment analysis indicated that the carcinogenicity of KRT17 can be attributed to cadherin binding, intermediate fibrocytoskeleton and epidermal development.

**Conclusion:** KRT17 may play an important role in the occurrence, development, and prognosis of malignant tumors. We provided a relatively comprehensive description of the carcinogenic role of KRT17 in different tumors for the first time.

## Introduction

Malignant tumors are a serious threat to human health and one of the major causes of death worldwide. In recent years, the morbidity and mortality rates of malignant tumors have increased significantly (Yao et al., 2019). Moreover, with the increase of population and poor lifestyle choices, the number of new cases and deaths related to malignant tumors is expected to increase rapidly (Torre et al., 2016). Early detection, early diagnosis, and treatment have become the goals for prevention and treatment of malignant tumors (Jemal et al., 2010). With the development of science and technology and the arrival of the era of precision medicine, searching for sensitive biomarkers and prognostic indicators of malignant tumors and exploring their molecular mechanisms are important for prevention and treatment. Given the complexity of malignancies, it is important to conduct pan-cancer expression analysis of any gene of interest and assess its correlation with clinical prognosis and its potential molecular mechanisms. With the rapid development of genomics, transcriptomics and proteomics, and the establishment of databases [The Gene Expression Omnibus (GEO), The Cancer Genome Atlas (TCGA), and The Human Protein Atlas (HPA)], the data can be conveniently accessed, allowing us to perform a pan-cancer analysis.

KRT17 is a triplet structure protein comprising 432 amino acids: a non-helical head (1–83), an α helical rod (84–392), and a non-helical tail domain (393–432) (Yang et al., 2019). The KRT17 gene is located on chromosome 17q21.2 (Kurokawa et al., 2011). The KRT17 is a multifunctional protein that regulates numerous cellular processes, including cell proliferation and growth (Depianto et al., 2010; Mikami et al., 2015). In addition, KRT17 can promote the release of inflammatory cytokines and promote the occurrence and development of tumors (Lo et al., 2010; Chung et al., 2015). Our team found that KRT17 expression was significantly different before and after cervical cancer radiotherapy when screening radiotherapy sensitivity genes (GSE6213). Considering that KRT17 may be a gene related to the radiotherapy sensitivity of cervical cancer, we assessed KRT17 and found that it was abnormally expressed in a variety of tumors after reviewing the literature. This abnormal expression is related to the occurrence, development, treatment, and prognosis of tumors (Ide et al., 2012; Ujiie et al., 2020). Moreover, despite the large number of clinical data, there is no pan-cancer evidence of a relationship between KRT17 and various tumor types. Therefore, we used databases or webservers such as TCGA, Tumor Immune Estimation Resource 2.0 (TIMER2.0), GEO, and Gene Expression Profiling Interactive Analysis 2 (GEPIA2) to conduct pan-cancer analysis of KRT17 and explore the potential molecular mechanisms by which it relates to the occurrence, development, and clinical prognosis of different cancer types.

## Materials and Methods

### Gene Expression Analysis

We searched the TIMER2.0 (http://timer.cistrome.org/) webservers in the Gene_DE KRT17 module input and found differences in KRT17 expression in tumor and normal tissues in TCGA database. There were no matched normal tissues in TCGA database. We obtained tumor tissues from the GEPIA2 (http://gepia2.cancer-pku.cn/) database and normal tissues from the Genotype-Tissue Expression (GTEx) database to assess the expression differences between the two tissue types. The *p* value cut-off was below 0.01, and the log fold change (logFC) cut-off was equal to 1. We selected “match TCGA normal and GTEx data” in the field of “match normal data.” With UALCAN tools (http://ualcan.path.uab.edu/index.html) and TCGA data analysis, we obtained KRT17 expression profiles for different tumor stages. UALCAN protein expression analysis (http://ualcan.path.uab.edu/home), *via* the “CPTAC analysis” module, was used to obtain the gene and protein expression profiles of KRT17 in tumor tissue and normal tissue.

### Analysis of Protein Expression

We entered “KRT17” into the “search” module of the HPA (https://www.proteinatlas.org/) network database, clicked the “search” button, and then selected the “tissue” and “pathology” modules to obtain KRT17 expression data for human tumor tissue samples and normal tissue samples and evaluate the protein expression of KRT17 on clinical specimen images. Staining reports included information of intensity, subcellular localization, single-cell variability, and antibodies. The staining intensity was classified into four categories (strong, moderate, weak, and negative) by using image capture and visualization techniques. The protein expression score was determined by the staining intensity from the immunohistochemistry (IHC) data and the proportion of stained cells as follows: negative–not detected; weak <25%–not detected; weak combined with either 25–75% or 75%–low; moderate <25%–low; moderate combined with either 25–75% or 75%–medium; strong <25%–medium; and strong combined with either 25–75% or 75%–high (Ta et al., 2021).

### Survival Prognosis Analysis

In the “start KM plotter for pan-cancer” list of the Kaplan–Meier Plotter (https://kmplot.com/analysis/), we obtained the overall survival (OS) and relapse-free survival (RFS) data of patients with various human malignancies sorted according to KRT17 expression. The median value was set as the cut-off. A Cox proportional hazards (PH) model was used to calculate the risk ratio. The log-rank sum test was used for hypothesis testing, and the OS plots and RFS plots were obtained *via* Kaplan–Meier Plotter survival analysis.

### Genetic Variation Analysis

In the cBioPortal (https://www.cbioportal.org/) dataset, we chose “TGCA extensive cancer atlas research” and searched for “KRT17” genetic variation characteristics. All the changes, mutational results, and copy number changes in TGCA tumors were reviewed in the cancer type summary. We also used the comparison/survival module to assess the differences in overall survival and disease-free, progression-free, and disease-free survival for cancer patients from TCGA database. Kaplan–Meier survival plots were generated by the log-rank sum test, and *p* < 0.05 was considered to indicate significance.

### Immune Infiltration Analysis

On the TIMER (http://timer.cistrome.org/) website “immune” template “gene expression” input (“KRT17”) and “immune infiltrates” [“CD8 + T cells”, “regulatory T cells” (Tregs), and “cancer-associated fibroblasts”] were selected to determine the relationships between KRT17 and tumor immune infiltration. The TIMER, CIBERSORT, CIBERSORT-abs, Quantiseq, Xcell, MCPCounter, and EPIC algorithms were used to estimate immune infiltration. The *p* values and bias correlation values were obtained by Spearman’s rank correlation test with purity adjustment. The data are presented as a scatter plot.

### Enrichment Analysis

First, the Search Tool for the Retrieval of Interacting Genes/Proteins (STRING) (https://string-db.org/) was used to screen 50 proteins that are experimentally verified to bind to KRT17. In the STRING webserver, we selected the column of “protein name”, entered “KRT17”, and selected “*Homo sapiens*”; the parameters were set as follows: "full network”, “evidence”, “experiments”, “low confidence (0.150)", and “no more than 50 interactors in the first shell”. After parameter setting, we continued to follow the instructions for the next step to obtain the binding proteins of KRT17. GEPIA2 was used for similar gene detection, and the first 200 similar genes were obtained. In addition, we used Jvenn, a Venn diagram viewer (http://bioinformatics.psb.ugent.be/webtools/Venn/), for cross analysis of KRT17 and its interacting genes, GEPIA2 was used for correlation analysis, Pearson’s correlation analysis was used for paired genes, and log2 transcripts per million (TPM) was applied to the dot plot to obtain the *p* value and correlation coefficient. In the Gene_cor module of the TIMER2.0 website, KRT17 was input as the gene of interest, and KRT5, KRT6a, KRT6b, KRT6c, and SFN gene expressions were analyzed. After execution, we generated a heatmap. The data included *p* values and bias correlation values obtained by purity-adjusted Spearman’s rank correlation test. For the Kyoto Encyclopedia of Genes and Genomes (KEGG) pathway analysis, we uploaded the data to the Database for Annotation, Visualization, and Discovery (DAVID) and then selected the settings “official_gene_symbol” and “*Homo sapiens*” to obtain the functionally annotated map data. The enriched pathways were analyzed using the “Tidyr” (https://cran.r-project.org/web/packages/tidyr/index.html) and “ggplot2” (https://Cran.r-project.org/web/packages/ggplot2/index.html) R language packages (version: version number: 3.6.2). In addition, we used the " clusterProfiler” (http://www.bioconductor.org/packages/release/bioc/html/clusterProfiler.html) R language package for the Gene Ontology (GO) enrichment analysis. *p* < 0.05 was considered to indicate statistical significance.

## Results

### Gene Expression Results

**FIGURE 1:**
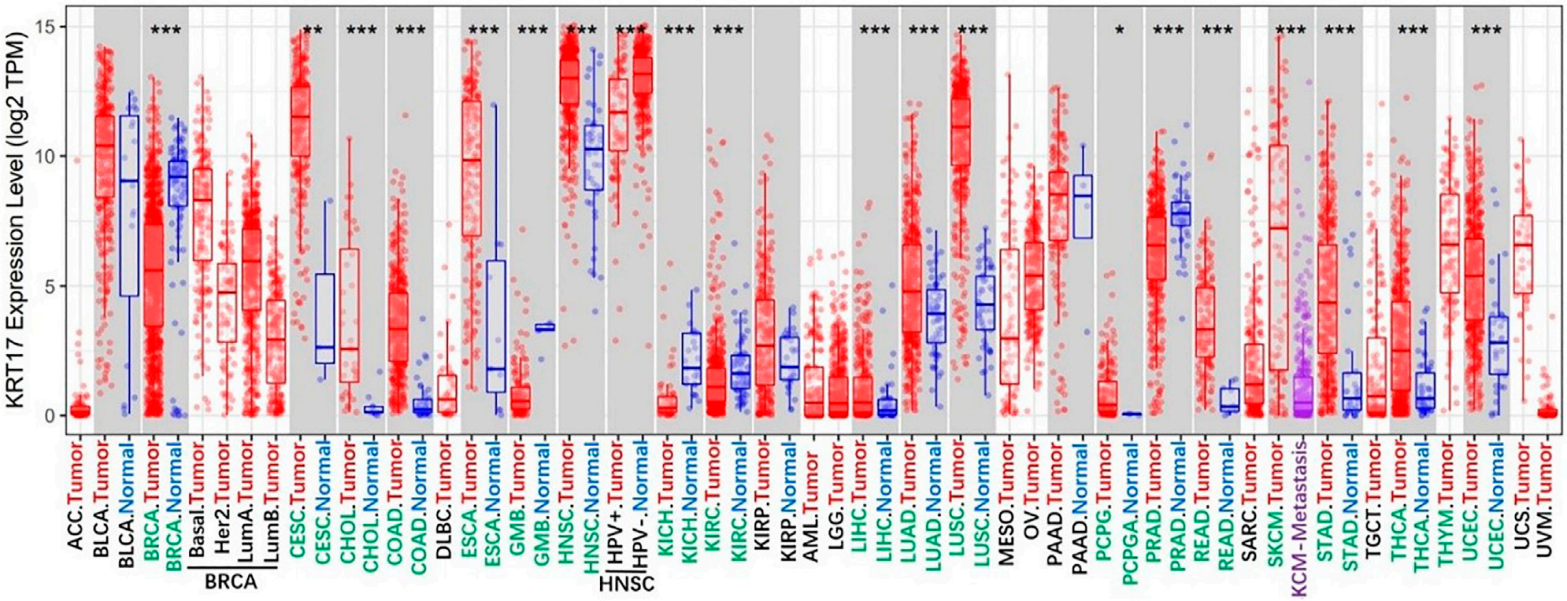
Expression status of KRT17 in various malignant tumors was analyzed through TIMER2; the figure showed that KRT17 expression in most malignant tumor tissues was higher than that in normal tissues, and it was statistically significant (∗*p* < 0.05; ∗∗*p* < 0.01; ∗∗∗*p* < 0.001) (TCGA dataset).

We determined the difference in the expression of KRT17 between various cancer types in TCGA database *via* the TIMER2.0 webserver (Figure 1). KRT17 expression was higher in cholangiocarcinoma (CHOL), colon adenocarcinoma (COAD), esophageal carcinoma (ESCA), glioblastoma multiforme (GBM), head and neck squamous cell carcinoma (HNSC), kidney renal clear cell carcinoma (KIRC), liver hepatocellular carcinoma (LIHC), lung squamous cell carcinoma (LUSC), lung adenocarcinoma (LUAD), prostate adenocarcinoma (PRAD), rectum adenocarcinoma (READ), skin cutaneous melanoma (SKCM), stomach adenocarcinoma (STAD), thyroid carcinoma (THCA), uterine corpus endometrial carcinoma (UCEC) (*p* < 0.001), cervical squamous cell carcinoma and endocervical adenocarcinoma (CESC) (*p* < 0.01), and pheochromocytoma and paraganglioma (PCPG) (*p* < 0.05) tissues than in normal tissues. In breast invasive carcinoma (BRCA) and kidney chromophobe (KICH), the expression of KRT17 was higher in normal tissues than in tumor tissues (*p* < 0.001). There was no difference in KRT17 expression between tumor and normal tissues in bladder urothelial carcinoma (BLCA), kidney renal papillary cell carcinoma (KIRP), and pancreatic adenocarcinoma (PAAD) (*p* > 0.05). TCGA, adrenocortical carcinoma (ACC), lymphoid neoplasm diffuse large B-cell lymphoma (DLBC), brain lower grade glioma (LGG), acute myeloid leukemia (AML), ovarian serous cystadenocarcinoma (OV), sarcoma (SARC), mesothelioma (MESO), testicular germ cell tumors (TGCT), thymoma (THYM), uterine carcinosarcoma (UCS), and uveal melanoma (UVM) datasets did not include matched normal tissues. Therefore, we looked for matched normal tissues in the GTEx database and used them as controls for tumor tissues in TCGA. We analyzed the previously mentioned tumors, and the expression of KRT17 in OV, THYM, and UCS tissues was higher than that in normal tissues (*p* < 0.05), while in LGG and TGCT, the expression of KRT17 in tumor tissues was lower than that in normal tissues (*p* < 0.05) (Supplementary Figure S1A). There was no significant difference in expression between ACC, DLBC, AML, and SARC tumor tissues and normal tissues. Unfortunately, there were no matched normal tissues for MESO and UVM.

**FIGURE 2:**
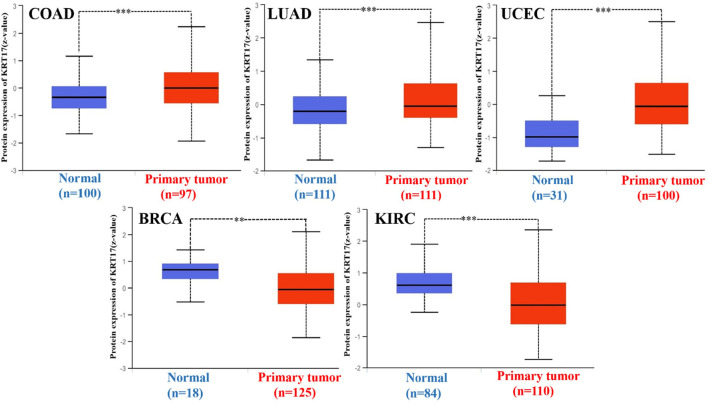
Expression of KRT17 in total protein of COAD, LUAD, UCEC, BRCA, and KIRC. The expression of KRT17 in COAD, LUAD and UCEC tumor tissues was higher than that in normal tissues, while in BRCA and KIRC, the expression of KRT17 in normal tissues was higher than that in tumor tissues (∗*p* < 0.05; ∗∗*p* < 0.01; ∗∗∗*p* < 0.001) (CPTAC dataset).

Next, we obtained the total protein expression data for KRT17 in BRCA, LUAD, COAD, UCEC, OV, and KIRC in tumor tissues and normal tissues in the CPTAC dataset, and the total protein expression data for the other cancers were not included in the CPTAC dataset. The total protein levels in COAD, LUAD, and UCEC tissues were higher than those in normal tissues, and the difference was statistically significant (*p* < 0.05); the levels in normal tissues were higher than those in tumor tissues in BRCA and KIRC, and the difference was statistically significant (*p* < 0.05); no significant difference was seen in OV (Figure 2). Since there were only six tumors with available total protein expression data in the CPTAC database, we obtained the expression data for BLCA, CESC, ESCA, HNSC, leukemia, LUSC, PAAD, and STAD from the oncomine database, and the differences in expression between these tumor tissues and their corresponding normal tissues were statistically significant (Supplementary Figure S1B).

We then analyzed the correlation between KRT17 expression in normal tissues and that in tumor tissues at different stages. The expression of KRT17 in CESC, HNSC, LUAD, LUSC, READ, and UCEC tissues of all stages was higher than that in normal tissues, and the differences were statistically significant (*p* < 0.05) (Supplementary Figure S1C); differences were also observed for BLCA (normal vs. Stage 1 and 2), COAD (normal vs. Stage 1, 3, and 4), ESCA (normal vs. Stage 2 and3), KIRP (normal vs. Stage 1 and 3) (LIHC (normal vs. Stage 1 and 2), and STAD (normal vs. Stage 2, 3, and 4), and THCA) (Normal vs. Stage 1 and 4). The expression of KRT17 in some stages was higher than that in normal tissues (Supplementary Figure S1D), while in BRCA, KICH and KIRC, the expression of KRT17 in each stage was lower than that in normal tissues, and the difference was statistically significant (*p* < 0.05) (Supplementary Figure S1E); no significant differences were found in other tumors (some of which did not have available data for matched normal tissue comparison; these included ACC, DLBC, MESO, UVM, OV, TGCT, and UCS) (Supplementary Figures S1F,H).

### Protein Expression Outcomes of KRT17 in Human Clinical Specimen

**FIGURE 3:**
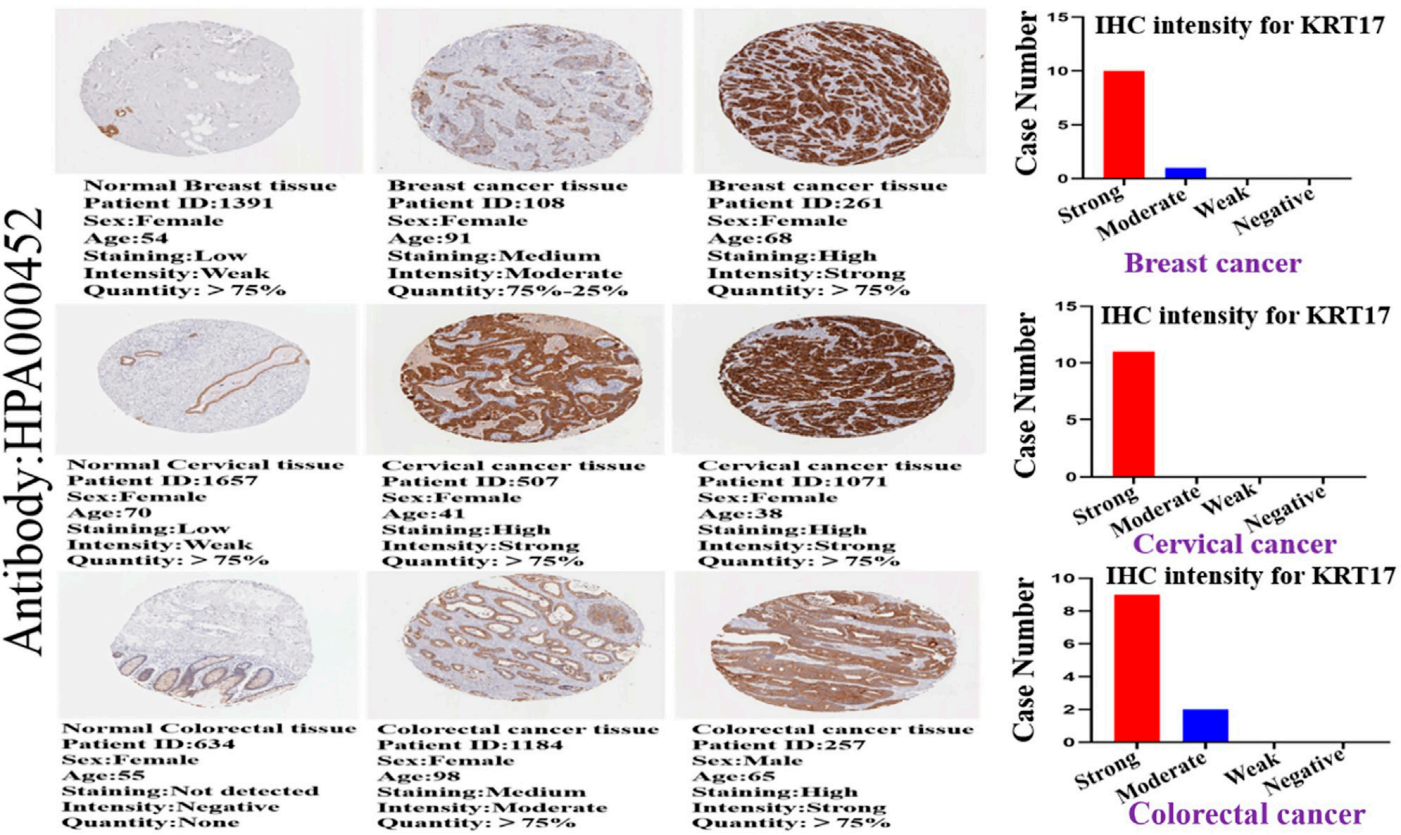
Immunohistochemical (IHC) images of normal and tumor tissues of KRT17 from patients with breast cancer, cervical cancer, and colorectal cancer, and the intensity of the IHC of KRT17. The bar graph shows the IHC intensity of KRT17 (breast cancer: 11 patients, cervical cancer: 11 patients, and colorectal cancer: 11 patients). All IHC images and patient information were derived from the HPA.

We investigated the protein expression of KRT17 in the HPA database, and we obtained IHC images of 19 types of cancer tissues and corresponding normal tissues; we also obtained corresponding clinicopathological parameters, such as patient ID, sex, age, and antibody. We found that KRT17 was overexpressed in BRCA, CESC, and colorectal cancer tissues versus normal tissues, and the difference was statistically significant (Figure 3). Moreover, KRT17 was overexpressed in BLCA, but there was no significant difference in the expression between cancer and normal tissues. In addition, the expression of KRT17 was low in glioma, LIHC, renal cancer, testicular cancer, and melanoma tissues, but significant differences were still observed between tumor and normal tissues. KRT17 was not expressed in lymphomas or normal lymph nodes. Other tumors showed moderate expression (Supplementary Figures S2A,B,C; Supplementary Table S1).

### Survival Outcomes Related to KRT17 Expression in Tumors

**FIGURE 4:**
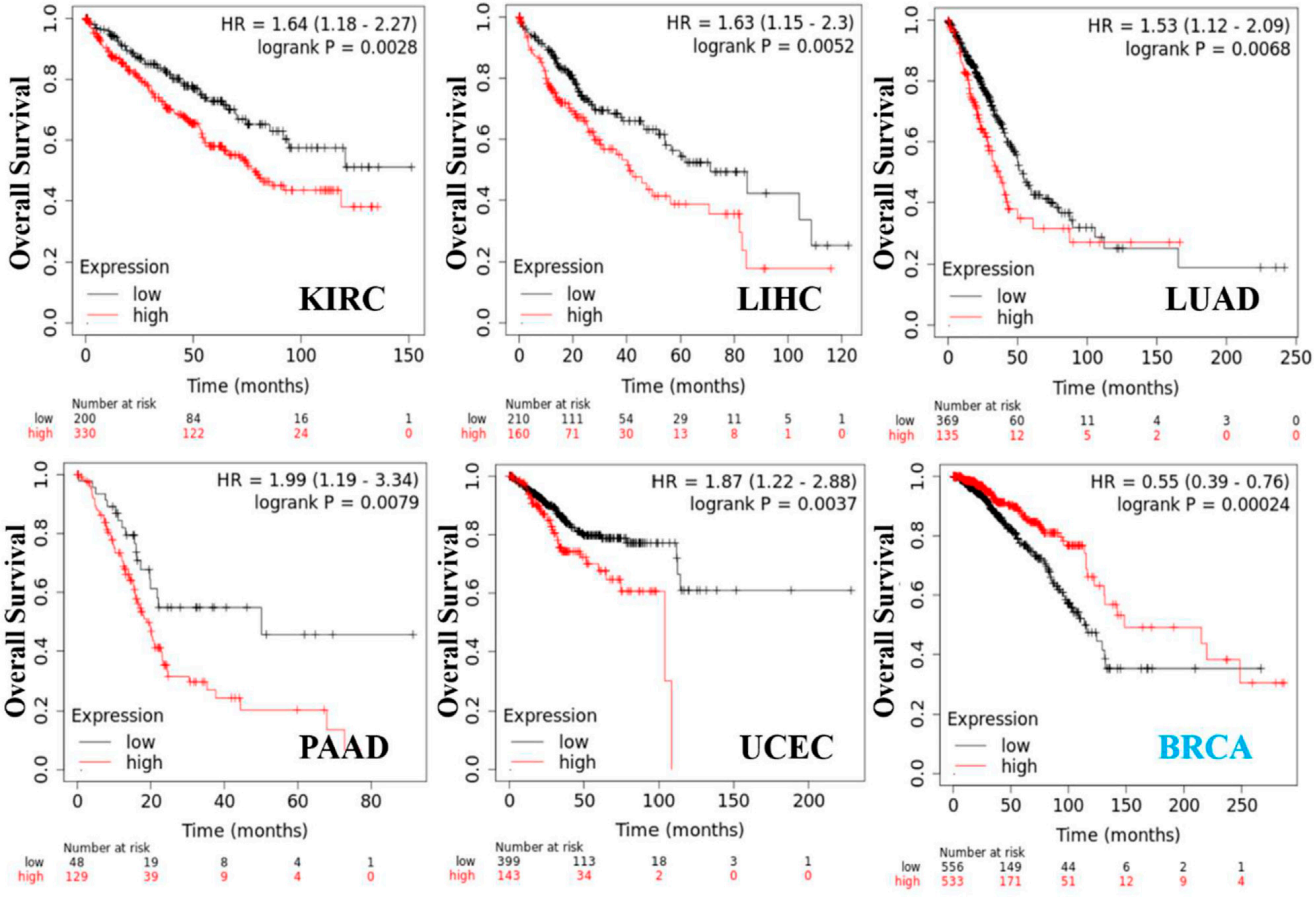
KRT17 expression in OS in KIRC, LIHC, LUAD, PAAD, UCEC, and BRCA. We obtained a relationship between KRT17 and survival prognosis of cancer from Kaplan–Meier Plotter. Kaplan–Meier curves were all positive. The solid red line represents the high expression of KRT17 in tumor tissues, and the solid black line represents the low expression of KRT17 in tumor tissues.

We obtained the survival prognosis information of patients sorted according to KRT17 expression level (high or low) for various cancer types through the Kaplan–Meier plotter. The correlation between KRT17 expression levels and survival prognosis of patients with different tumors was studied (Figure 4). The OS of KIRC, LIHC, LUAD, PAAD, and UCEC patients with high expression of KRT17 was lower than that of those with low expression of KRT17 (*p* < 0.05); however, the OS rate in BRCA was higher for patients with high expression of KRT17 than for those with low expression of KRT17 (*p* < 0.05). High expression of KRT17 was associated with favorable RFS in THCA, KRIC, and UCEC, while unfavorable RFS was associated with BLCA and PAAD (*p* < 0.05) (Supplementary Figure S3).

### Genetic Variation Results for KRT17

**FIGURE 5:**
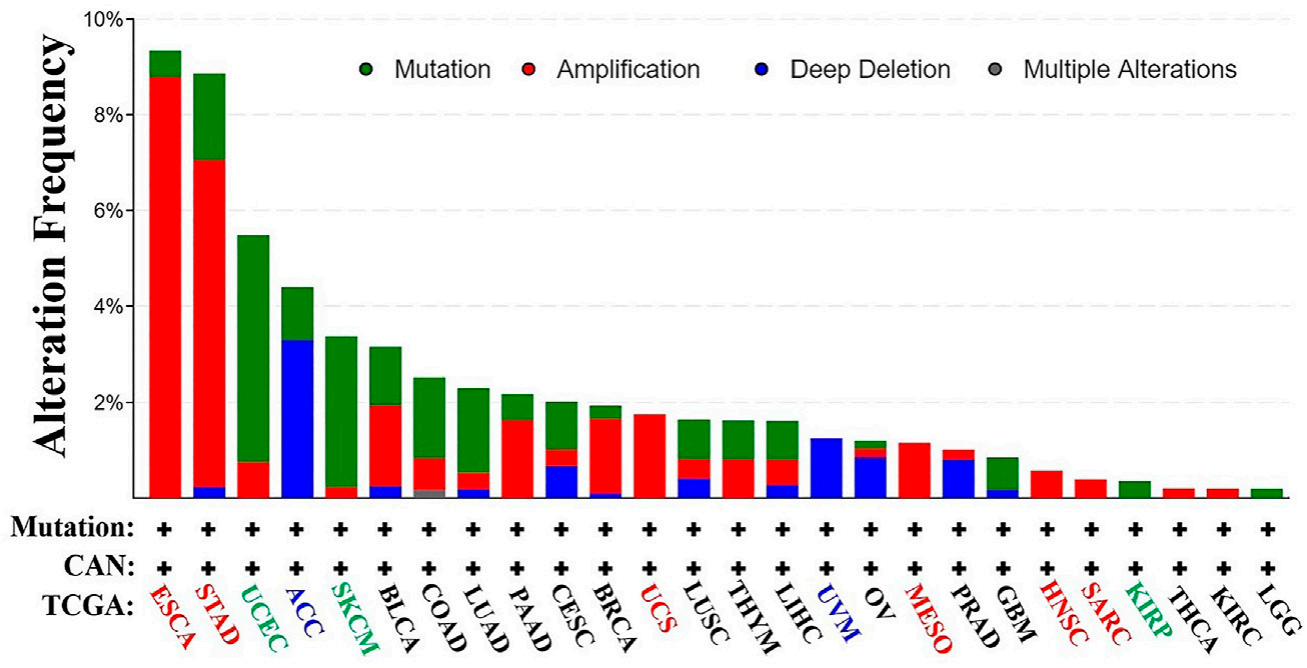
Alteration frequency of KRT17 in a variety of malignancies. The figure showed that KRT17 was the main type of amplification in esophageal cancer, the main type of mutation in UCEC, and the main type of deep deletion in ACC (TCGA dataset).

**FIGURE 6:**
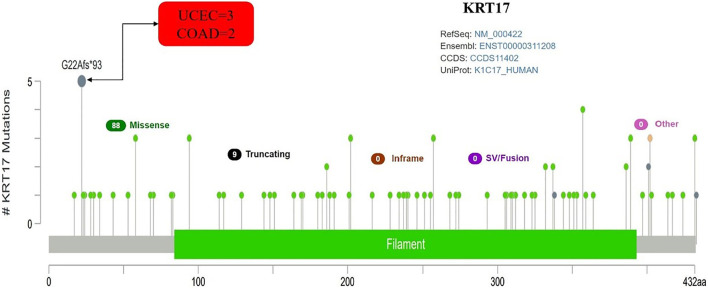
Mutation site and number of cases of KRT17. G22Afs∗93 was the site with the highest mutation frequency. There were three cases in UCEC and two cases in COAD (TCGA dataset).

**FIGURE 7:**
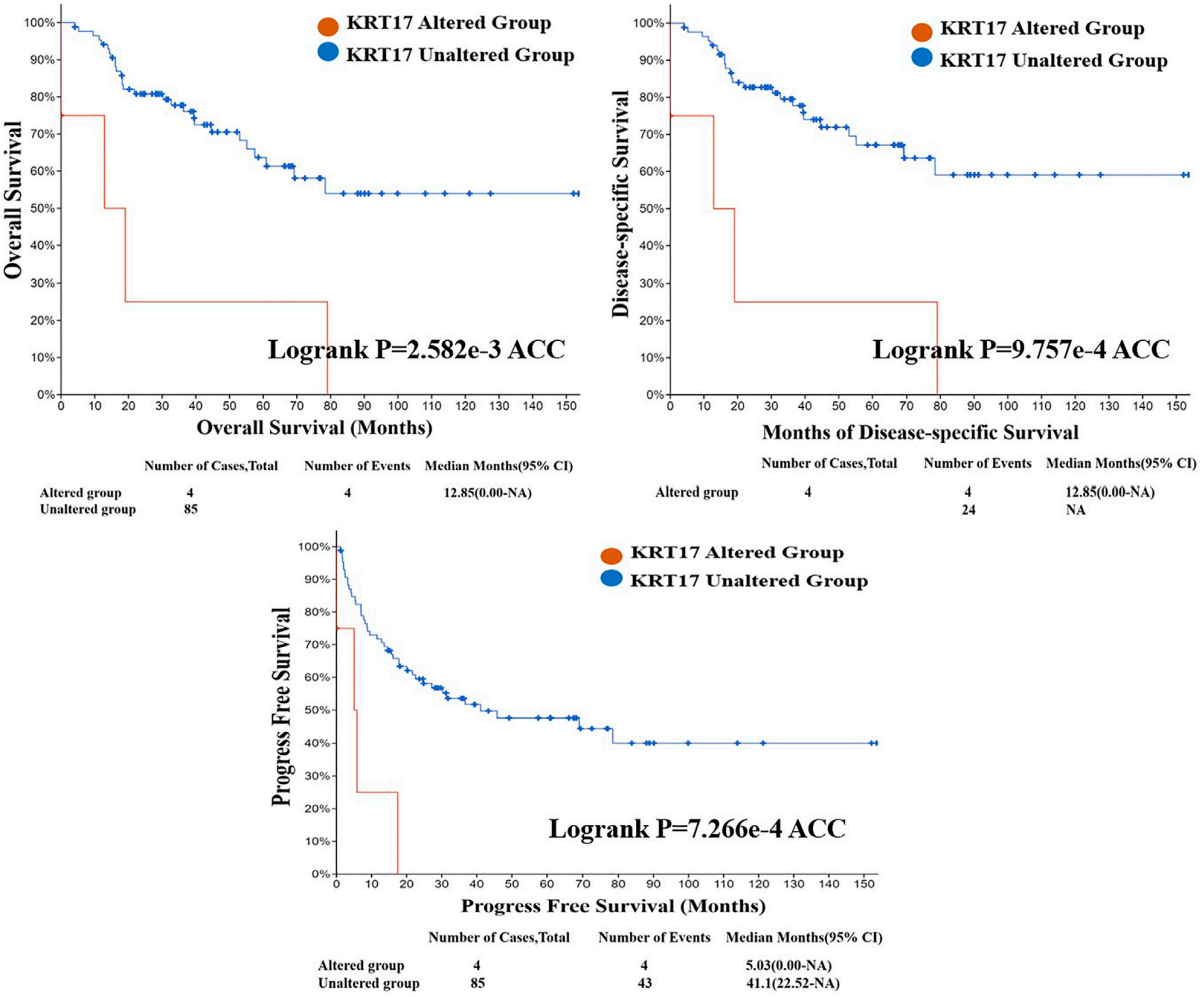
Correlation between KRT17 mutation status and overall survival (OS), disease-specific survival (DSS), and progression-free survival (PFS) of ACC. The solid red line represents the KRT17-altered group in tumor tissues, and the dash-dotted blue line represents KRT17-unaltered group in tumor tissues (TCGA dataset).

From TCGA database, we learned the expression state of KRT17 gene genetic variation in different tumors (Figure 5). Among all the variation expression states, the amplification type was associated with the highest expression, and the amplification rate was approximately 9% and was the highest in ESCA and STAD. Mutation was the dominant type of genetic variation in UCEC, with a mutation frequency of approximately 4%. In addition, KRT17 gene copy deletion was found in both ACC and MESO. Furthermore, we learned about the type, locus, and number of cases of genetic variation in KRT17. The main type of genetic variation in KRT17 was frameshift mutation. In addition, we noted that the KRT17 protein exhibited a change from glycine (G) to alanine (A) at site 22 in three UCEC cases and two COAD cases (Figure 6). We also explored the correlation between KRT17 gene mutation and survival prognosis of patients with different tumors. Compared with patients without KRT17 mutations, the overall survival (*P* = 2.582e-3), disease-specific (*P* = 9.757e-4), and progression-free (*P* = 7.266e-4) rates for ACC were lower for the KRT17 mutant group than for the non-mutant group (Figure 7). In addition, KRT17 mutations were found to be associated with survival in CESC (progression-free survival), THCA (progression-free survival and disease-free), and THYM (overall survival and survival). However, in SKCM (progression-free survival), the prognosis of the KRT17 mutant group was superior to that of the KRT17 mutant group (Supplementary Figure S4).

### Results of Immune Cell Infiltration Analysis

**FIGURE 8:**
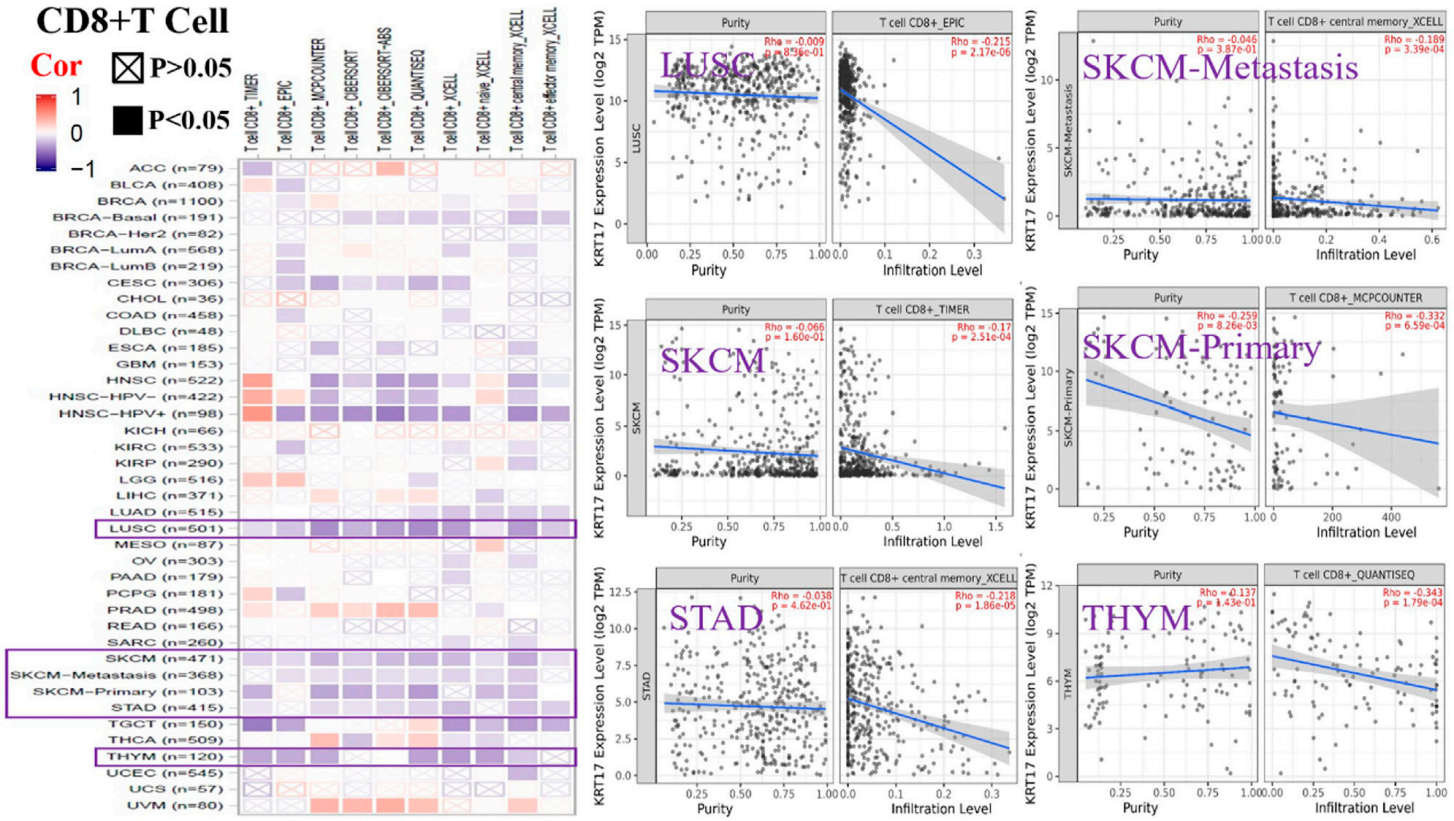
Correlation between KRT17 expression and immune infiltration of CD8+T cells. In LUSC, SKCM, SKCM-metastasis, SKCM-primary, STAD and THYM and KRT17 expression was negatively correlated with CD8+T cell expression (TCGA dataset).

**FIGURE 9:**
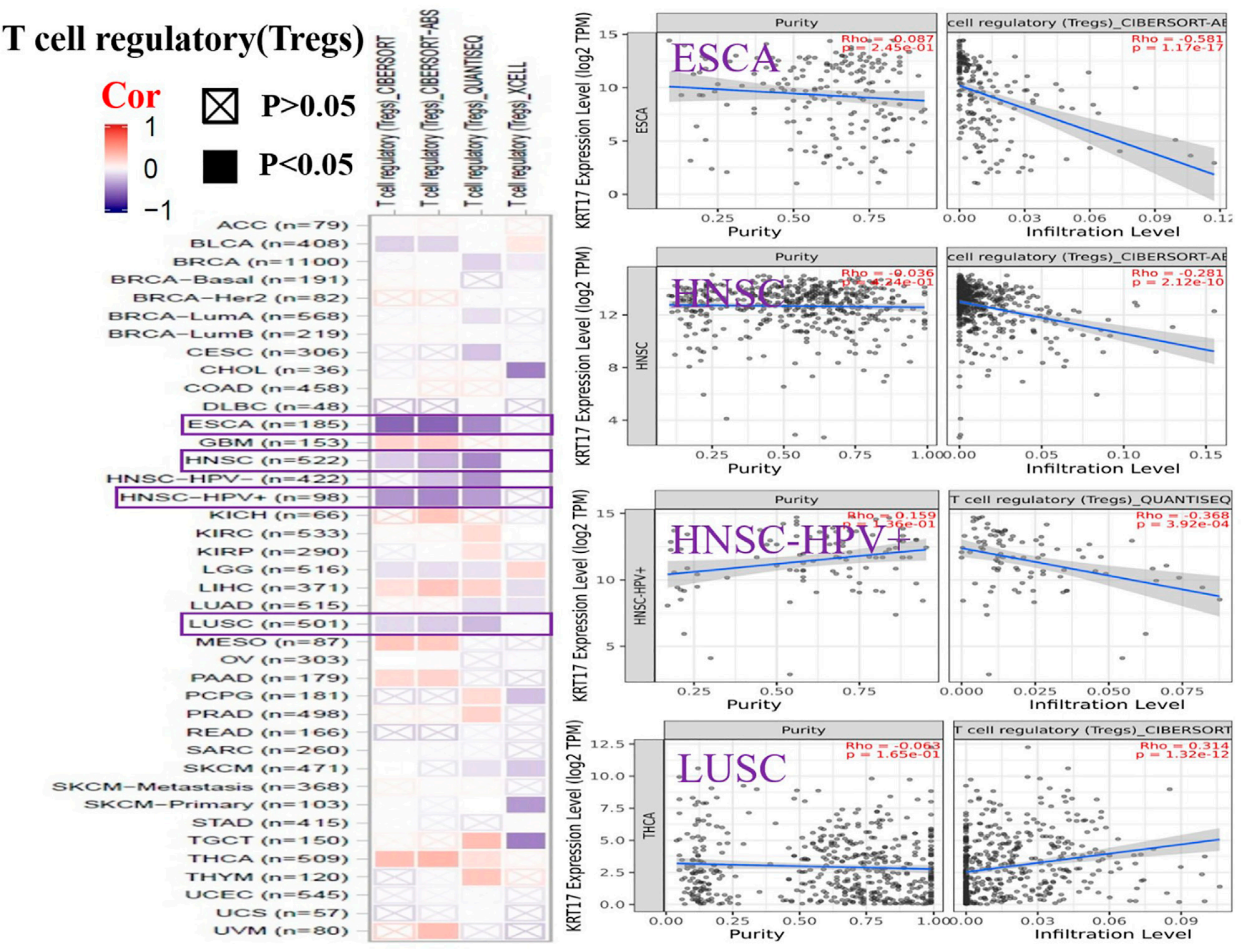
Correlation between KRT17 expression and immune infiltration of Tregs cells. In ESCA, HNSC, and HNSC-HPV+ and LUSC, KRT17 expression was negatively correlated with Treg expression (TCGA dataset).

**FIGURE 10:**
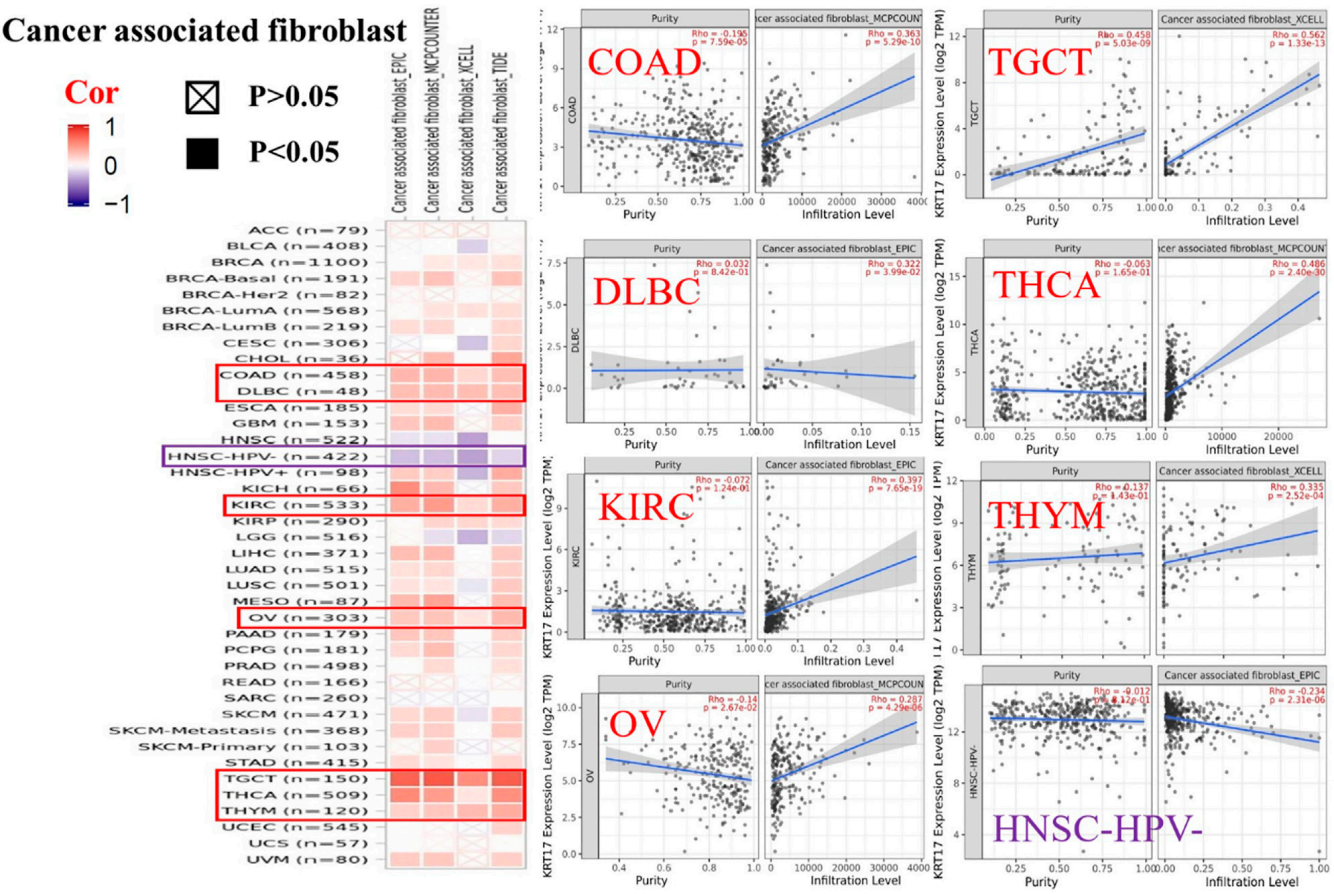
Correlation between KRT17 expression and immune infiltration of cancer-associated fibroblasts. In THYM, TGCT, KIRC, COAD, THCA, OV, and DLBC, KRT17 expression was positively correlated with cancer-associated fibroblasts expression; however, in HNSC-HPV-, KRT17 expression was negatively correlated with cancer-associated fibroblast cells (TCGA dataset).

To further clarify the relationship between KRT17 and tumor-infiltrating immune cells, we used the TIMER, CIBERSORT, CIBERSORT-abs, Quantiseq, Xcell, MCPCounter, and EPIC methods to investigate the potential relationship between the level of infiltration of different immune cells and the expression of the KRT17 gene in different types of cancer in TCGA database. We found that KRT17 expression and macrophage infiltration were positively correlated in LIHC, THCA, THYM, and UVM (Supplementary Figure S5A). KRT17 expression and CD8+T cell infiltration were negatively correlated in LUSC, SKCM, SKCM-metatasis, SKCM-primary, STAD, and THYM (Figure 8). KRT17 expression was negatively associated with Treg infiltration in ESCA, HNSC, HNSC- human papilloma virus positive (HNSC-HPV+), and LUSC (Figure 9) but positively associated with Treg infiltration in THCA (Supplementary Figure S5B). In addition, we found that KRT17 expression was positively correlated with cancer-associated fibroblast infiltration in COAD, DLBC, KIRC, OV, TGCT, THCA, and THYM and negatively correlated with cancer-associated fibroblast infiltration in HNSC (Figure 10).

### Results of Enrichment Analysis

**FIGURE 11:**
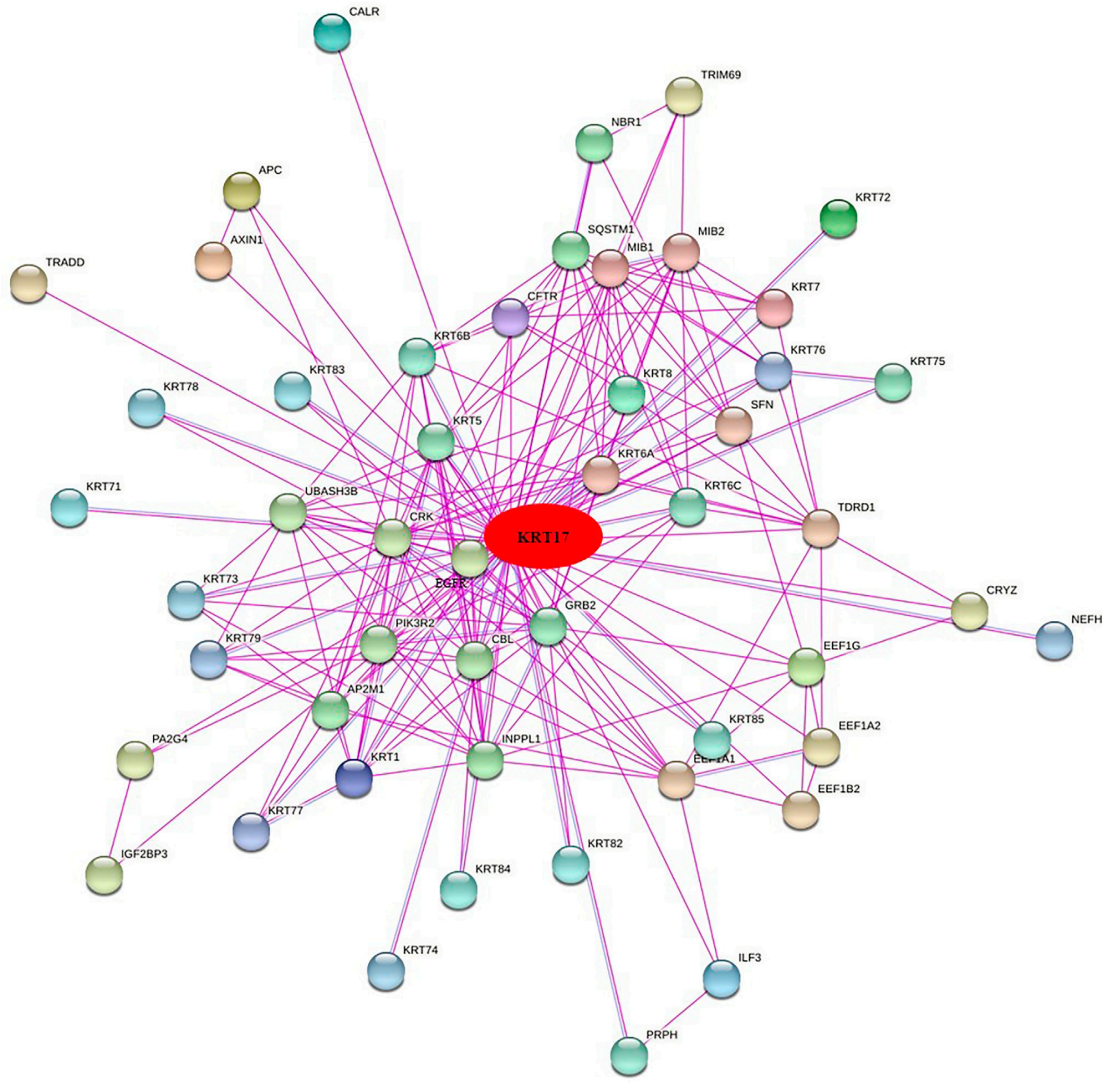
Fifty experiments confirmed the binding proteins interacting with KRT17 and their interaction networks.

**FIGURE 12:**
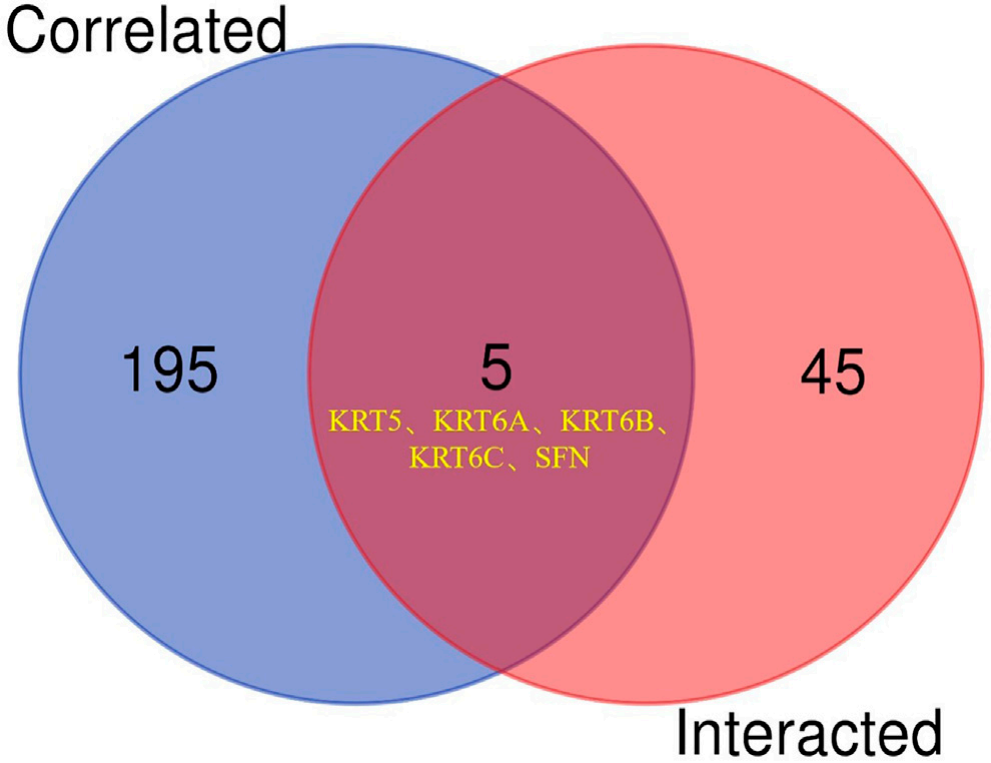
Cross analysis of KRT17 binding protein and related genes. An intersection analysis of the abovementioned two groups showed five common members, namely, KRT5, KRT6A, KRT6B, KRT6C and SFN.

**FIGURE 13:**
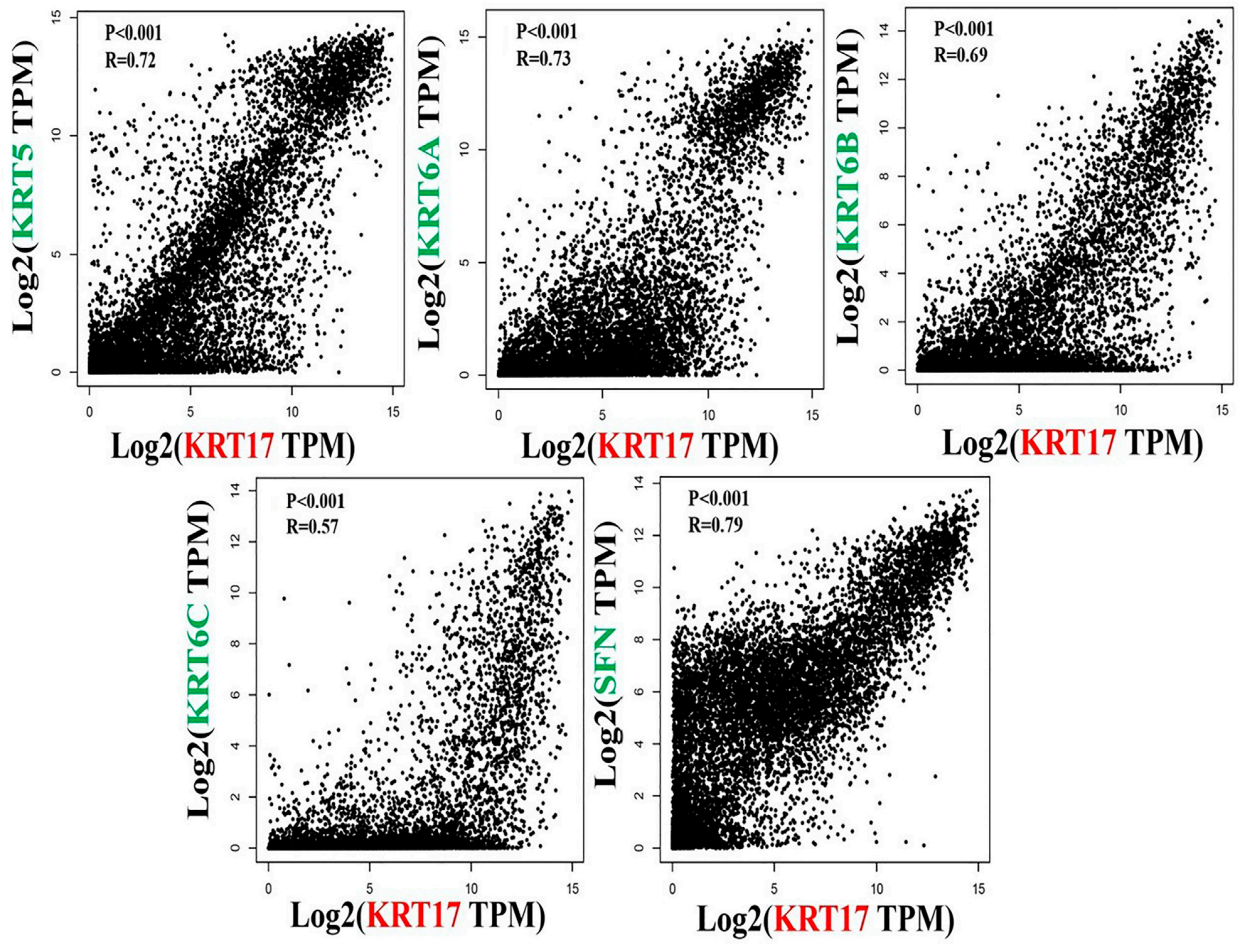
Correlation between KRT17 and KRT5, KRT6a, KRT6b, KRT6c and SFN. The results showed that the expression of KRT17 was positively correlated with the expression of KRT5, KRT6a, KRT6b, KRT6c and SFN.

**FIGURE 14:**
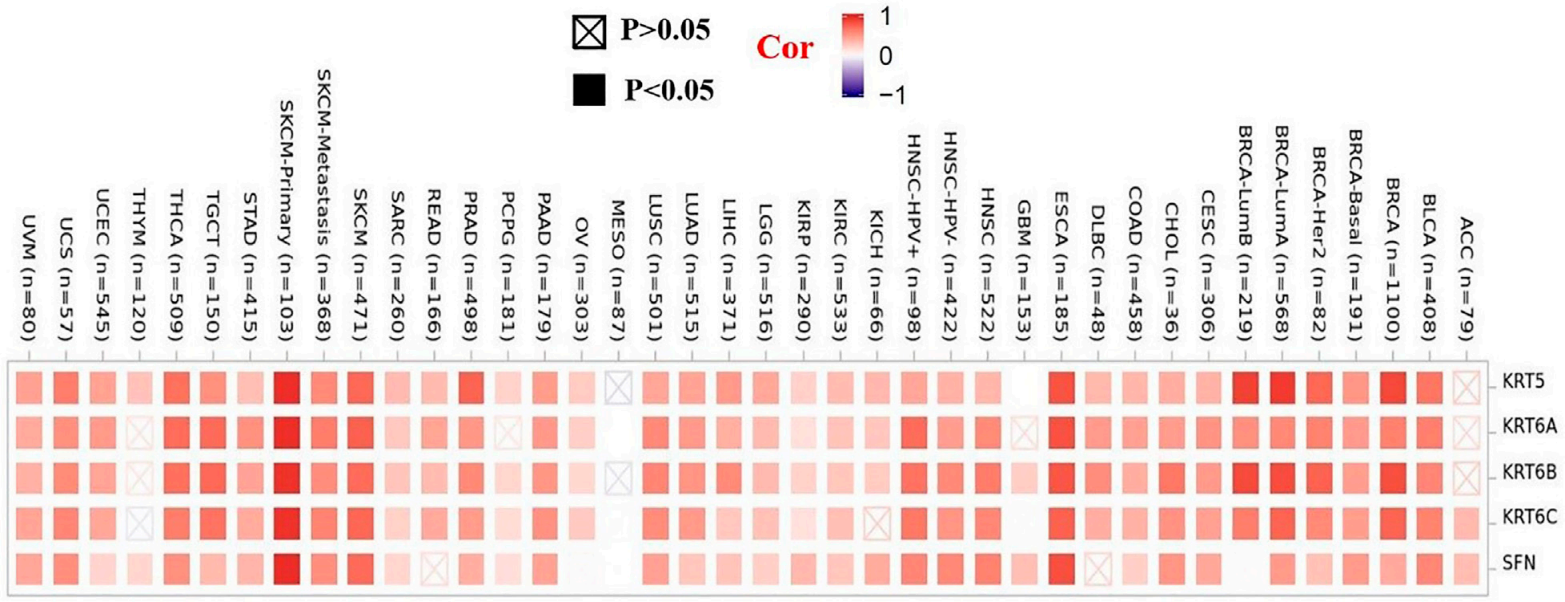
Heatmap data between KRT17 and KRT5, KRT6A, KRT6B, KRT6C, and SFN. The heatmap results showed that the expression of KRT17 was positively correlated with the expression of KRT5, KRT6a, KRT6b, KRT6c, and SFN.

To further clarify the mechanism by which KRT17 mediated the pathogenesis of tumors, we screened the binding proteins interacting with KRT17 and the genes related to KRT17 expression. Using the STRING tool, we obtained 50 experimentally confirmed KRT17 binding proteins and generated an interaction network of these proteins (Figure 11). We combined the tumor expression data of TCGA and GTEx with the GEPIA2 tool to obtain the top 200 genes related to KRT17 expression. To further screen genes, we analyzed the intersection between 50 binding proteins interacting with KRT17 and the top 200 genes related to KRT17 expression. Five genes (KRT5, KRT6A, KRT6B, KRT6C, and SFN) were obtained (Figure 12). Through the GEPIA2 tool, we obtained the correlation between the expression of KRT17 and that of KRT5, KRT6a, KRT6b, KRT6c, and SFN, and the results showed that the expression of KRT17 was positively correlated with that of these five genes (Figure 13). In addition, we used TIMER2.0 to obtain a heatmap between KRT17 and KRT5, KRT6A, KRT6B, KRT6C, and SFN, and the corresponding heatmap results also showed that KRT17 was positively correlated with the abovementioned five genes in most tumors (Figure 14).

**FIGURE 15:**
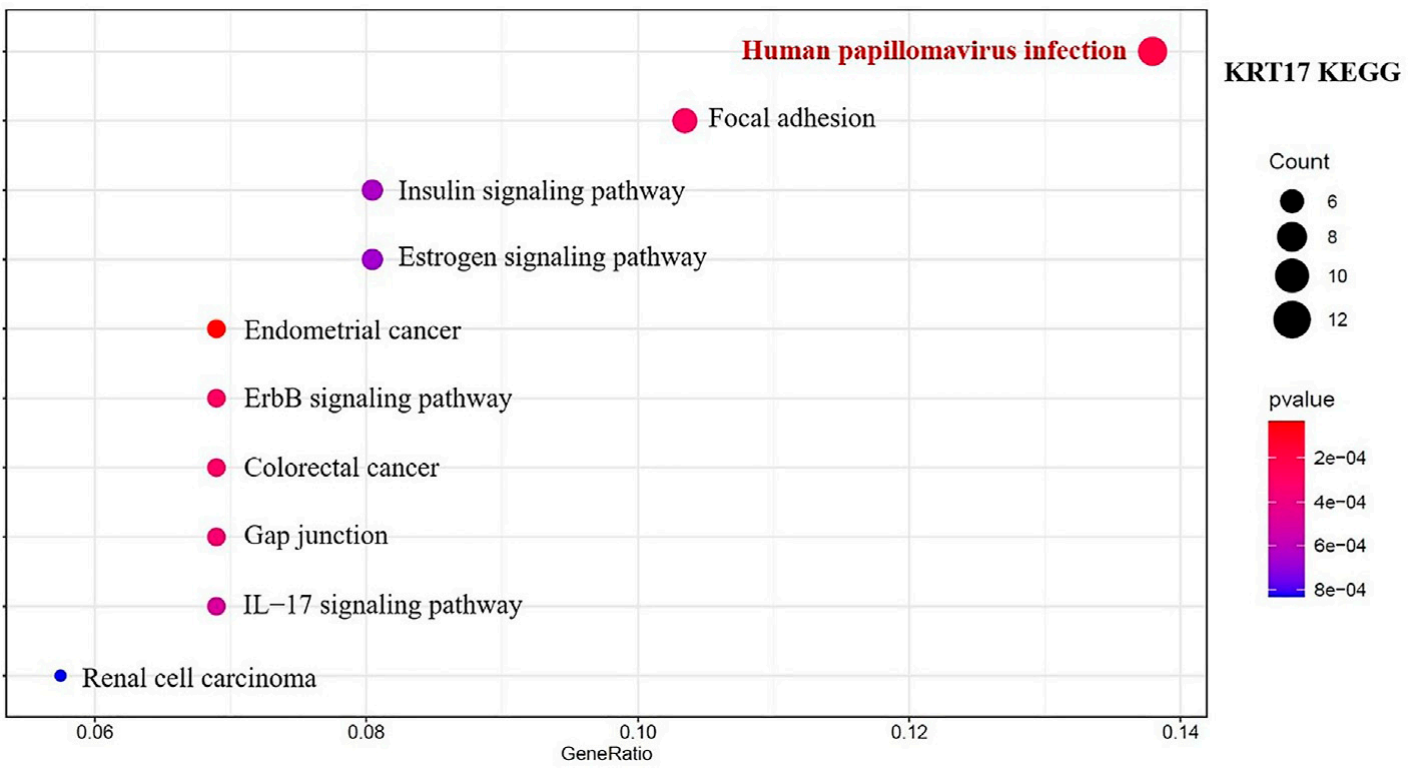
Results of KEGG pathway analysis of KRT17 interaction binding and expression-related genes.

We combined 50 binding proteins interacting with KRT17 with two datasets of the top 200 genes related to KRT17 expression and carried out KEGG and GO enrichment analyses. Through KEGG analysis, we learned that KRT17 may play a role in the pathogenesis of tumors mainly through HPV infection (Figure 15). GO enrichment analysis showed that these two sets of genes were mainly involved in cadherin binding, intermediate cytoskeleton filament formation, and epidermal development (Supplementary Figures S6A–C).

## Discussion

Keratin is a member of the intermediate filament superfamily that makes up the cytoskeleton and is encoded by 54 evolutionarily conserved genes. According to gene substructure and nucleotide sequence homology, keratin can be divided into two types: 28 type I acidic proteins and 26 type II basic proteins (Jacob et al., 2018). Keratin 17 (KRT17) belongs to the type I intermediate family and is an intermediate filament of the cytoskeleton, involved in structural support, metabolism, and various developmental processes (McGowan and Coulombe, 1998; Pan et al., 2013). KRT17 is mainly found in epithelial appendages, such as hair follicles, sebaceous glands, and other glands (Kurokawa et al., 2011). KRT17 is not expressed in the epidermis of normal skin, but its expression can be induced under stress conditions, such as skin injury (Kim et al., 2006) and virus infection (Proby et al., 1993). KRT17, as an intermediate filament, has long been thought to play a role in the cytoplasm. However, recent studies have shown that due to the presence of nuclear localization signals and nuclear output signals, KRT17 can move both inside and outside the nucleus, suggesting that KRT17 may regulate additional cellular processes (Hobbs et al., 2016a). In recent years, there have been an increasing number of reports on the relationship between KRT17 and malignant tumors, especially the functional association between tumors (Chivu-Economescu et al., 2017; Liu et al., 2018; Chen et al., 2020; Yan et al., 2020). However, it is still unclear whether KRT17 has a common molecular mechanism that plays a role in the occurrence and development of different tumors. To date, there have been no reports of KRT17 in pan-cancer studies. Therefore, we analyzed KRT17 in 33 different tumors by employing TCGA, TIMER2.0, GEO, GEPIA2, and HPA databases or websites and analyzed and summarized its molecular characteristics such as gene expression and gene mutation and its associations with clinical prognosis and immune infiltration. KRT17 is highly expressed in most malignant tumors, suggesting that KRT17 may play a role as an oncogene in cancers. Among the total proteins, KRT17 expression was higher in COAD, LUAD, and UCEC than in normal tissues, while KRT17 expression was higher in BRCA and KIRC normal tissues than in tumor tissues. In addition, we analyzed the expression of KRT17 protein in human tumor tissues and normal tissues in HPA and found that KRT17 was overexpressed in breast cancer, cervical cancer, colorectal cancer, and bladder cancer, but there was no statistical significance between bladder cancer tissues and normal tissues. The mRNA and total protein expressions of KRT17 in normal tissues were higher than those in tumor tissues, while the immunohistochemical results showed that KRT17 was highly expressed in tumor tissues, which may be related to the selected specimens, tumor heterogeneity, and detection technology, so it is necessary to continue to expand clinical samples for research. In glioma, liver cancer, kidney cancer, testicular cancer, and melanoma, KRT17 expression is low but higher than that in corresponding normal tissues. However, KRT17 is moderately expressed in thyroid cancer, lung cancer, gastric cancer, prostate cancer, ovarian cancer, head and neck cancer, and endometrial cancer. These results were consistent with the mRNA expression results, suggesting that KRT17 may play an oncogenic role in the occurrence and development of malignant tumors (except for BRCA). The results of the *in vitro* experiments were consistent with the previously mentioned results. KRT17 was found to be highly expressed in cervical cancer, esophageal cancer, lung cancer, gastric cancer, and colorectal cancer cell lines. KRT17 knockout was also found to inhibit cell proliferation and migration and increase sensitivity to cisplatin chemotherapy in cervical cancer cells (Escobar-Hoyos et al., 2015). Similarly, in esophageal cancer, lung cancer, gastric cancer, and colorectal cancer, KRT17 knockout can inhibit cancer cell proliferation, migration, invasion, and colony formation and induce apoptosis (Chivu-Economescu et al., 2017; Wang et al., 2019; Liu et al., 2020; Ujiie et al., 2020). The previously mentioned results confirmed that the knockout of KRT17 inhibited the growth, migration, and invasion of tumor cells, while the overexpression of KRT17 had the opposite effect, suggesting that KRT17 may be involved in the occurrence and development of malignant tumors and play an oncogenic role in malignant tumors.

Subsequently, the expression of KRT17 at various tumor pathological stages was analyzed, and the expression of KRT17 in most tumors at various pathological stages was higher than that in normal tissues, while the expression in normal tissues was higher than that in BRCA, KICH, and KIRC tissues at different stages. According to GEPIA2, high expression of KRT17 in most tumors is associated with poor prognosis, while high expression of KRT17 is associated with a favorable prognosis in BRCA. These results indicate that KRT17 may play a role as a tumor suppressor gene in BRCA; this finding could also be explained by other reasons. The prognosis of BRCA is related not only to stage and treatment but also to molecular type. In BRCA, the prognosis of triple-negative breast cancer is worse than that of other types of breast cancer. Merkin et al. studied tissues from 164 breast cancer patients, among which 82% (28/34) of triple-negative [estrogen receptor [ER]/progesterone receptor/human epidermal growth factor receptor-2 (HER2) negative] breast cancers showed positive KRT17 expression. The positive expression rate of KRT17 in non-triple-negative breast cancer was 46% (52/112). High expression of KRT17 was associated with reduced 5-year DFS in patients with advanced cancer. Studies have shown that high KRT17 expression is associated with triple-negative status and reduced survival (Merkin et al., 2017). However, the Kaplan–Meier plotter indicated that high expression of KRT17 has good prognostic implications in breast cancer. Considering that the proportion of triple-negative breast cancer cases included in TCGA is small (approximately 14% of all breast cancer cases), high expression of KRT17 seems to indicate a good prognosis. Similarly, in lung cancer, high expression of KRT17 is not associated with OS in LUSC (Wang et al., 2019), while in LUAD, high expression of KRT17 is associated with survival (Liu et al., 2018), indicating that the expression status and clinical prognosis implications of KRT17 may be related to pathological classification. However, only 239 patients were included in the two studies, so a larger sample size is needed to confirm the role of KRT17 in survival outcomes in patients with different types of lung cancer. In addition, in KIRC and UCEC, high expression of KRT17 was negatively correlated with OS, while in RFS, high expression of KRT17 was positively correlated with RFS. Considering that KRT17 may play different roles in the development of tumor diseases, how it affects the survival and mechanism of the disease still needs to be further explored.

Our study also showed that the expression state of KRT17 genetic variations in various tumors, with gene amplification, gene mutation, and gene deletion as the main variation type. Analysis of TCGA–ACC dataset revealed that the KRT17 mutation status of ACC was associated with OS, DSS, and PFS, In ESCA, STAD, and UCEC, a correlation was not found, indicating that tumor development and clinical outcomes are not simply influenced by mutation status but rather by a complex genetic process. Among human diseases, mutations in KRT17 have been reported to be associated with congenital thyroid disease. Among KRT17 mutations, c.275A > G missense mutations that cause asparagine to be replaced by serine (Asn92Ser) are the most common (Cogulu et al., 2009; Ofaiche et al., 2014). In UCEC and COAD, we found that KRT17 translation from G (glycine) to A (alanine) (Gly22Ala) at site 22 is the most common; the change at this point may be the main reason for the occurrence and development of malignant tumors caused by KRT17, but the specific molecular mechanism is still not clear and needs further study. Cancer-related immunology is complex and poorly understood, partly reflected in the diversity of immune responses and the spatial and temporal heterogeneity of developing tumors (Melero et al., 2014). Studies have shown that during the occurrence of cervical cancer, the expression of some inflammatory cytokines and immune cytokines in tumors is significantly dependent on KRT17 (Hobbs et al., 2016b). In addition, KRT17 can activate different macrophage populations and subtypes (such as M1 and M2) through interferon γ, tumor necrosis factor-α and interleukin 10, which play an important role in tumor proliferation and differentiation (Sica and Mantovani, 2012). Therefore, we used the Quantiseq, Xcell, Mpcounter, and EPIC methods to analyze the relationship between KRT17 expression and the infiltration of macrophages, CD8+T cells, Tregs, and cancer-related fibroblasts. There was a positive correlation between KRT17 expression and macrophage infiltration in LIHC, THCA, THYM, and UVM, but no positive correlation was found in cervical cancer. Considering that the expression of interferon γ, tumor necrosis factor-α and interleukin 10 may be different in different patients with cervical cancer, KRT17 may have little effect on stimulating the production of macrophages through the abovementioned factors. KRT17 expression was negatively associated with Treg infiltration T-regulatory cells in ESCA, HNSC, HNSC- human papilloma virus positive (HNSC-HPV+), and LUSC but positively associated with Treg infiltration in THCA. THCA is an endocrine gland. Considering that the high expression of Tregs may be related to hormones secreted by endocrine glands, studies have shown that Tregs infiltration is greater in THCA and the increase of Tregs tissue infiltration is positively correlated with advanced disease (Gogali et al., 2012). Therefore, the expression of Tregs may be used as a biomarker and prognostic indicator of THCA. This also indicates that there are multiple ways in which KRT17 acts in malignant tumors. In addition, we proposed the relationship between KRT17 and CD8+T cells, Tregs, and cancer-related fibroblasts for the first time, suggesting that KRT17 and immune cells are involved in the formation and development of tumors, However, the specific mechanism of action is still unclear and needs further study.

Next, GO enrichment analysis was performed on the binding proteins interacting with KRT17 and the genes related to KRT17 expression. Through the analysis, we learned that the expression of KRT17 was positively correlated with the expression of KRT5, KRT6a, KRT6b, KRT6c, and SFN. We also found that HPV infection, the estrogen signaling pathway, cadherin binding, and intermediate cytoskeleton filament formation may affect KRT17-mediated tumor pathogenesis and development. Recent studies have shown that KRT17 has a variety of mechanisms of action in malignant tumors and can inhibit tumor cell proliferation, migration, and invasion by regulating the Akt/mTOR pathway, glucose uptake (Khanom et al., 2016), Wnt signaling pathway, epithelial–mesenchymal transition (EMT) (Wang et al., 2019), and mTOR/S6K1 signaling pathway (Li et al., 2020). This suggests that the mechanism of action of KRT17 in malignant tumors is complex and diverse, that there may be multiple mechanisms in one tumor, and that multiple tumors may share similar mechanisms (Li et al., 2019; Liu et al., 2020; Zeng et al., 2020).

To summarize, the correlation between KRT17 expression and clinical prognosis, genetic variation and immune cell infiltration were analyzed for the first time. These results are helpful for understanding the role of KRT17 in tumorigenesis and development and exploring its potential clinical application value, providing useful information for KRT17 research and drug development.

## Data Availability

The original contributions presented in the study are included in the article/Supplementary Material, further inquiries can be directed to the corresponding author.
