# Supplementary material for: Analysis of the Expression and Role of Keratin 17 in Human Tumors
Source: Front Genet. 2022 May 12;13:801698. doi: 10.3389/fgene.2022.801698 (PMC9133940; doi:10.3389/fgene.2022.801698)
Supplement: Supplementary file 1 [file DataSheet1.docx]

**The expression and role analysis of Kertin 17(KRT17)**

**in human tumors**

**Supplementary Material**


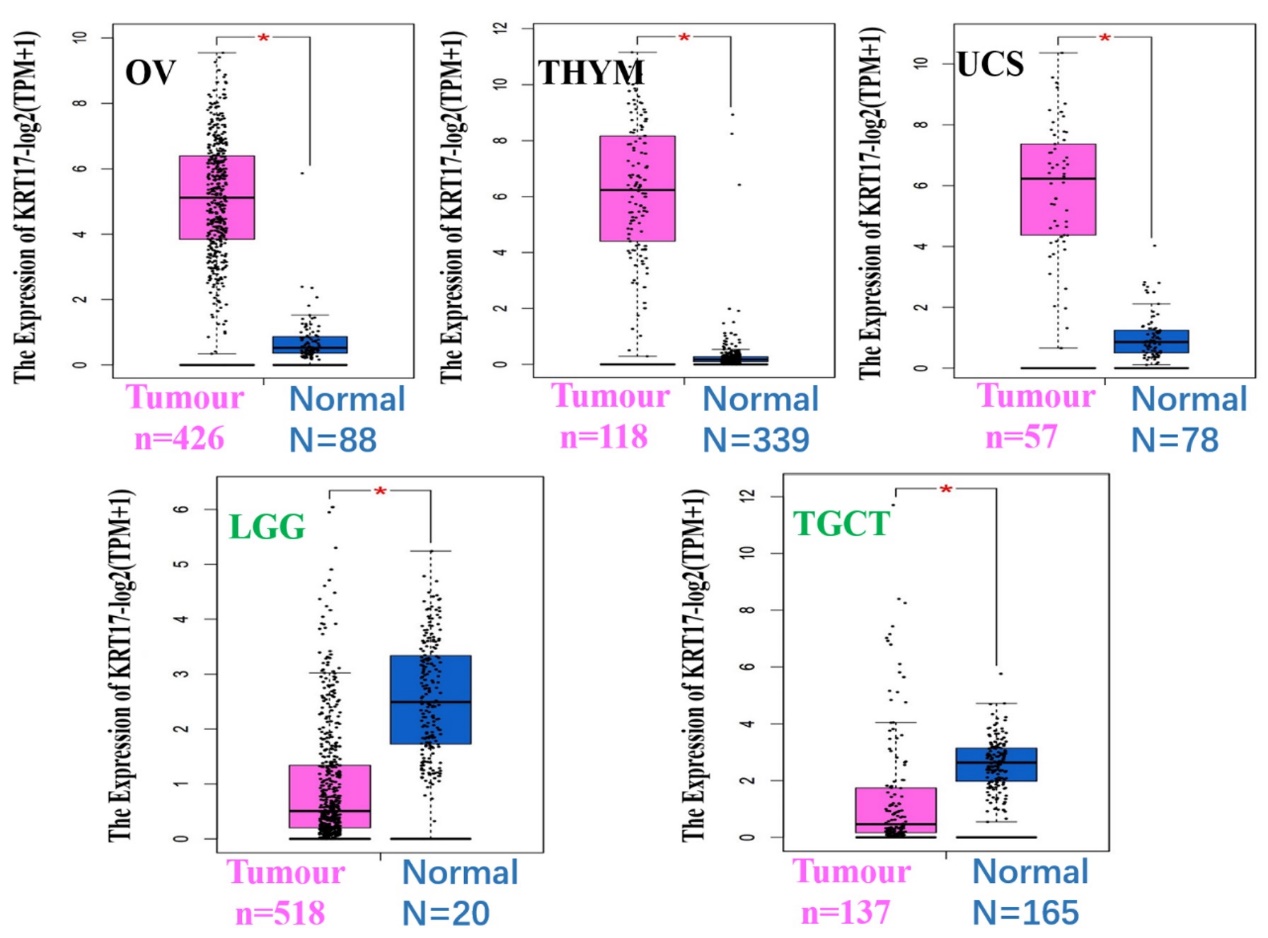


**Figure S1a.** The expression status of KRT17 in OV, THYM, UCS, LGG and TGCT. The expression of KRT17 in OV, THYM and UCS tumor tissues was higher than that in normal tissues, while in LGG and TGCT, the expression of KRT17 was lower than that in normal tissues. The differences were statistically significant.（*P<0 .05; **P<0 .01; ***P< 0.001）（TCGA+GTEx dataset）.


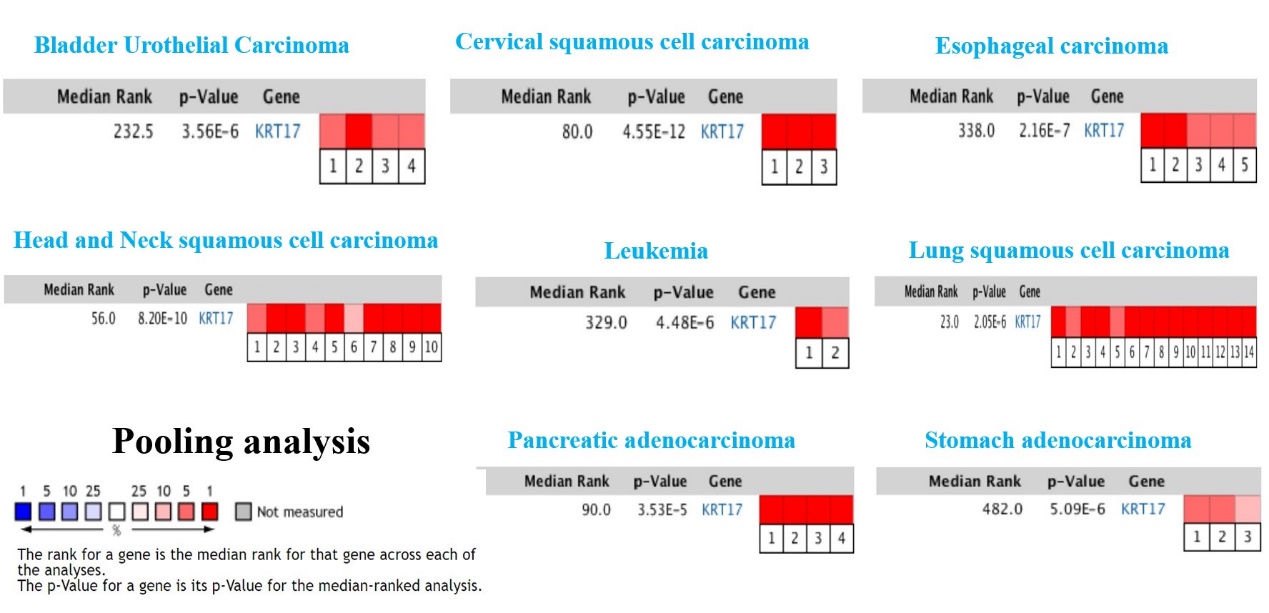


**Figure S1b**. The expression of total proteins of KRT17 in BLCA, CESC, ESCA, HNSC, Leukemia, LUSC, PAAD and STAD, In addition, KRT17 was highly expressed in these tumor tissues, and the differences were statistically significant. (Oncomine dataset).


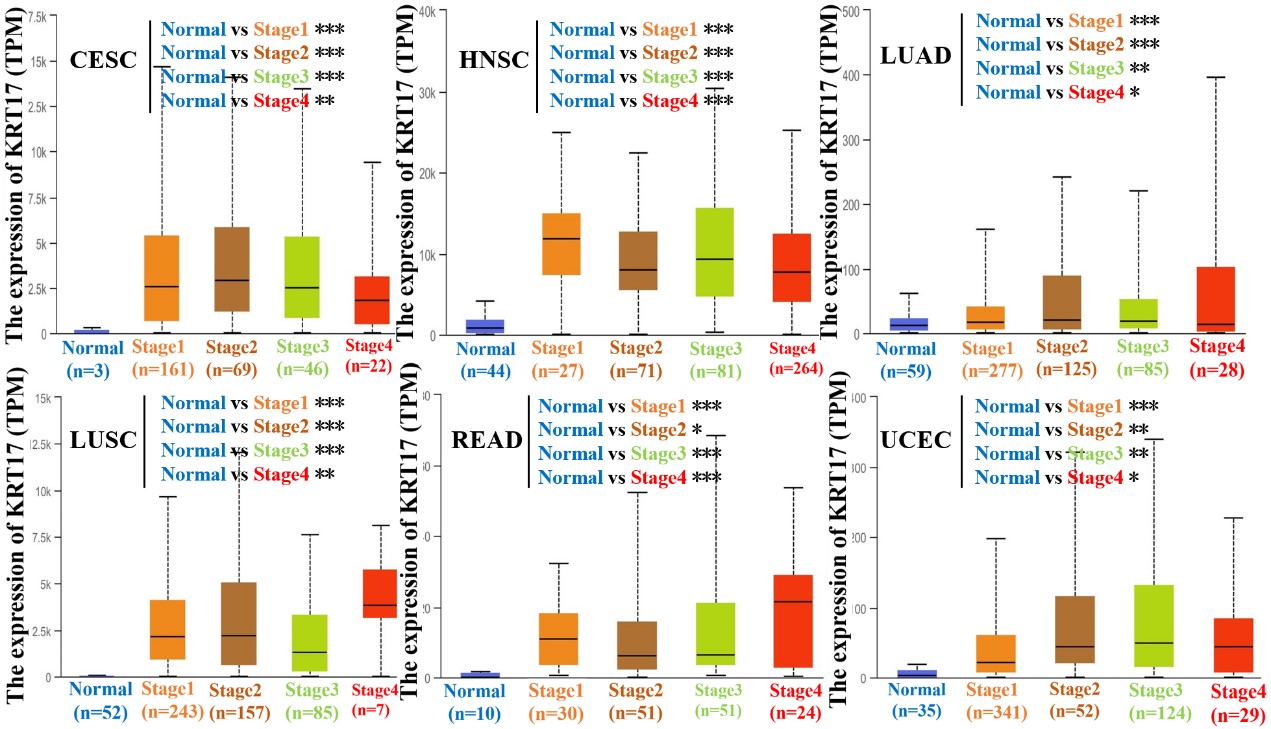


**Figure S1c.** The expression of KRT17 in different tumor pathological stages and normal tissues. In CESC, HNSC, LUAD, LUSC, READ, and UCEC, KRT17 expression in stage 1, 2, 3, and 4 was higher than that in normal tissues, and the differences were statistically significant.（*P<0 .05; **P<0 .01; ***P< 0.001）（TCGA dataset）.


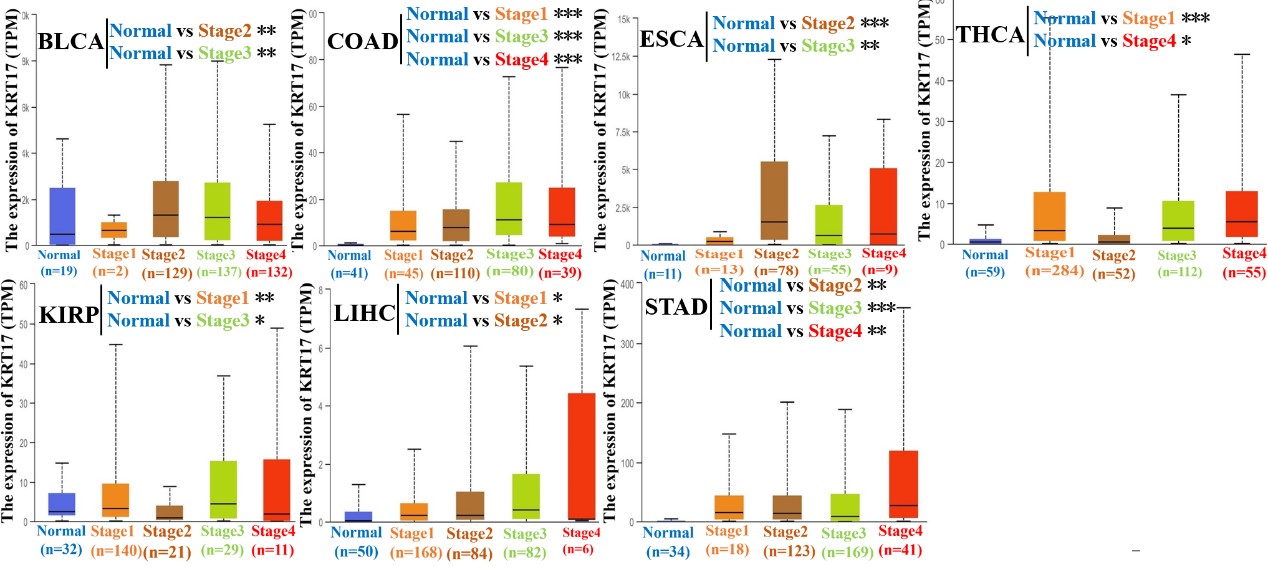


**Figure S1d.** The expression of KRT17 in different tumor stages and normal tissues. In BLCA (stage2and 3), COAD(stage1,3and 4), ESCA (stage2 and 3), THCA(stage1 and 4), KIRP (stage1 and 3), LIHC(stage 1 and 2) and STAD(stage 2,3 and 4), KRT17 expression was higher than that in normal tissues, and the difference was statistically significant.（*P<0 .05; **P<0 .01; ***P< 0.001）（TCGA dataset）.


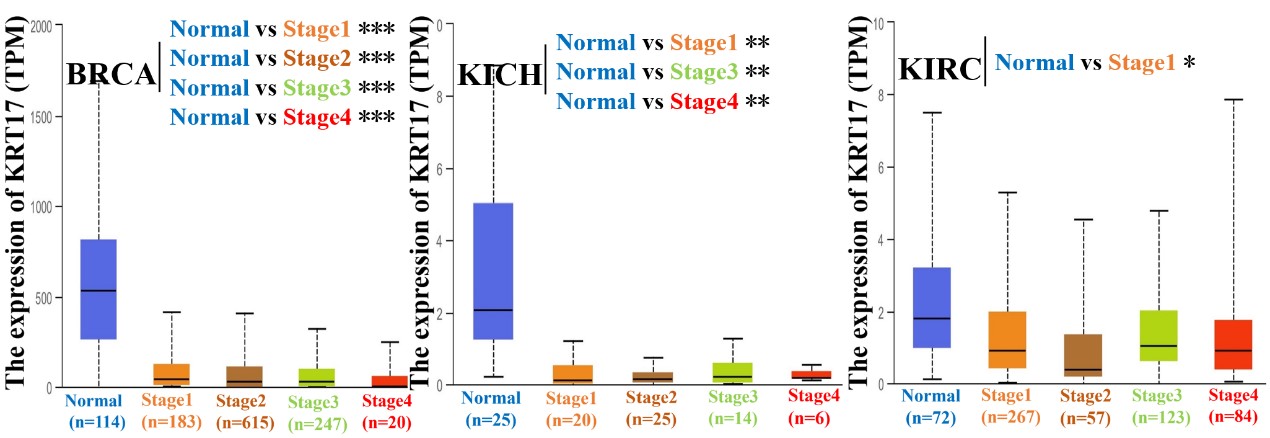


**Figure S1e.** The expression of KRT17 in different tumors, different stages and normal tissues (KRT17 is highly expressed in normal tissues)（*P<0 .05; **P<0 .01; ***P< 0.001）（TCGA dataset）.


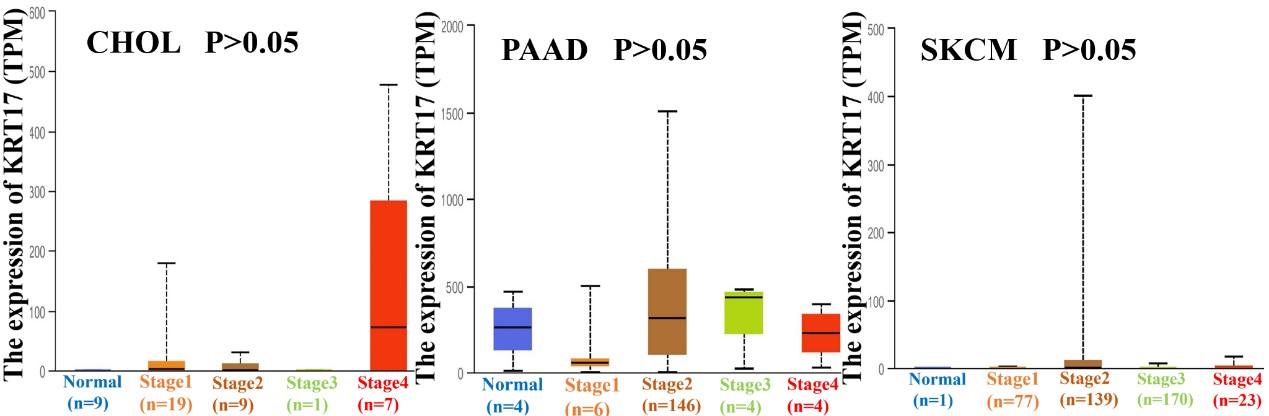


**Figure S1f.** The expression of KRT17 in different tumor stages and normal tissues (no statistical significance)（TCGA dataset）.


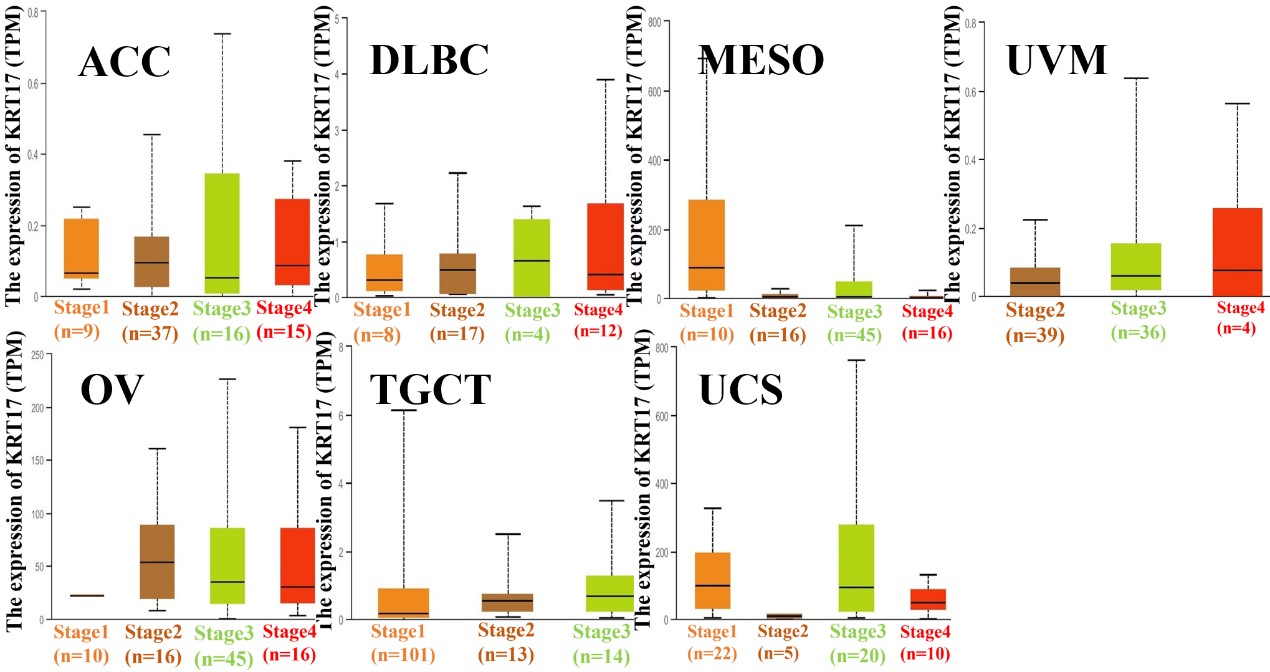


**Figure S1h.** The expression of KRT17 in different tumors at different stages (no matched normal tissues)（TCGA dataset）.


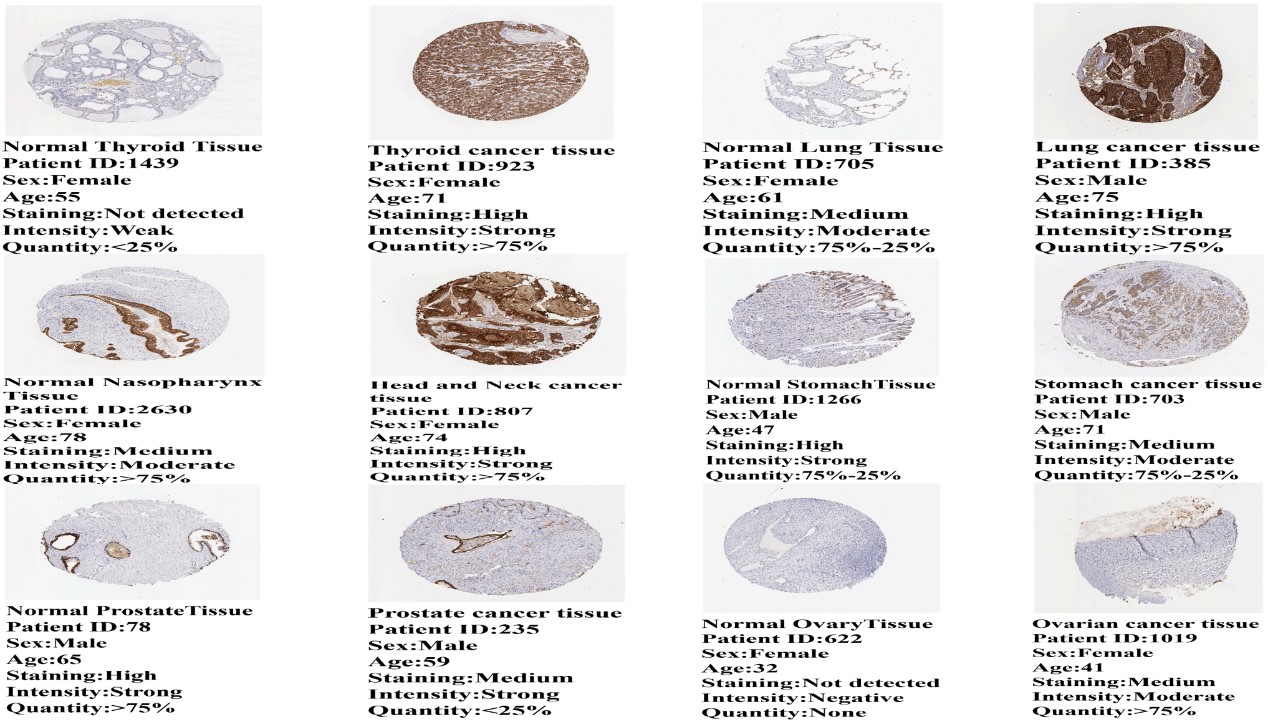


Figure S2a. Immunohistochemical (IHC) images of normal and tumor tissues of KRT17 from patients with Thyroid cancer, Lung cancer, Head and Neck cancer, Stomach cancer,Prostate cancer and Ovarian cancer. All IHC images and patient information were derived from the HPA.


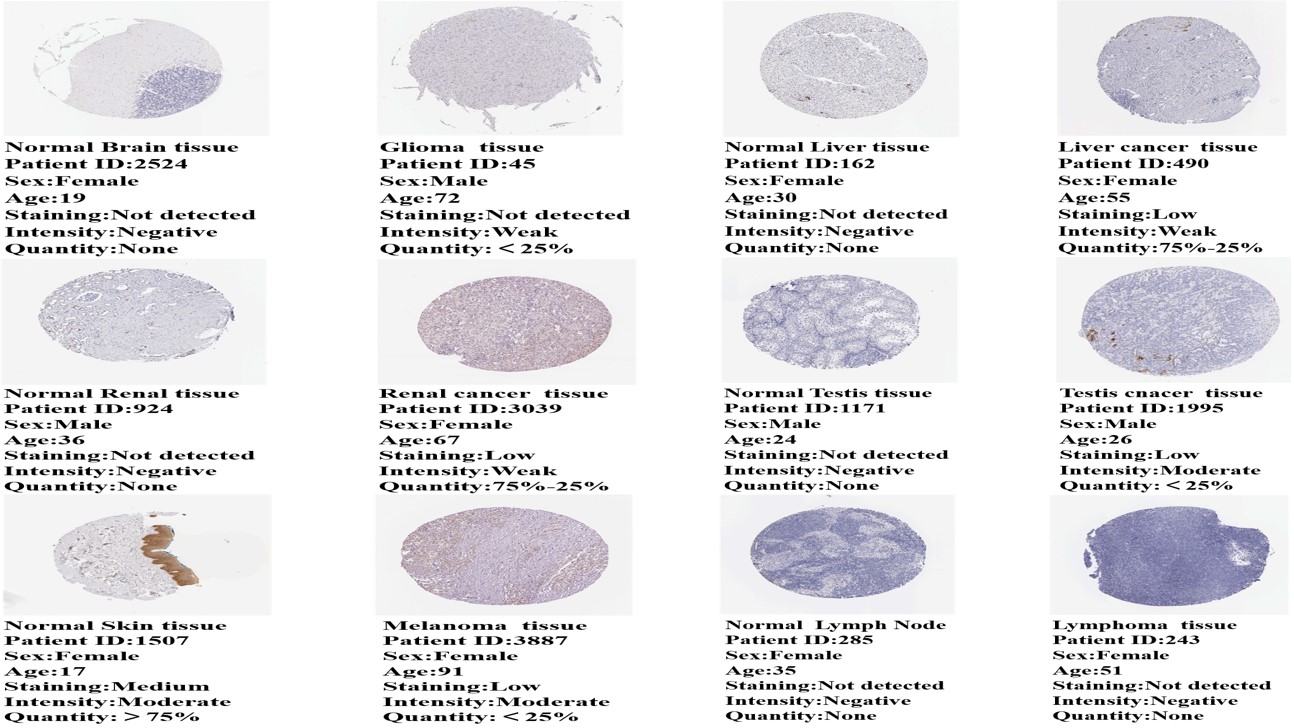


Figure S2b. IHC images of normal and tumor tissues of KRT17 from patients with Glioma, Liver cancer, Renal cancer, Testis cancer,Melanoma and Lymphoma. All IHC images and patient information were derived from the HPA.


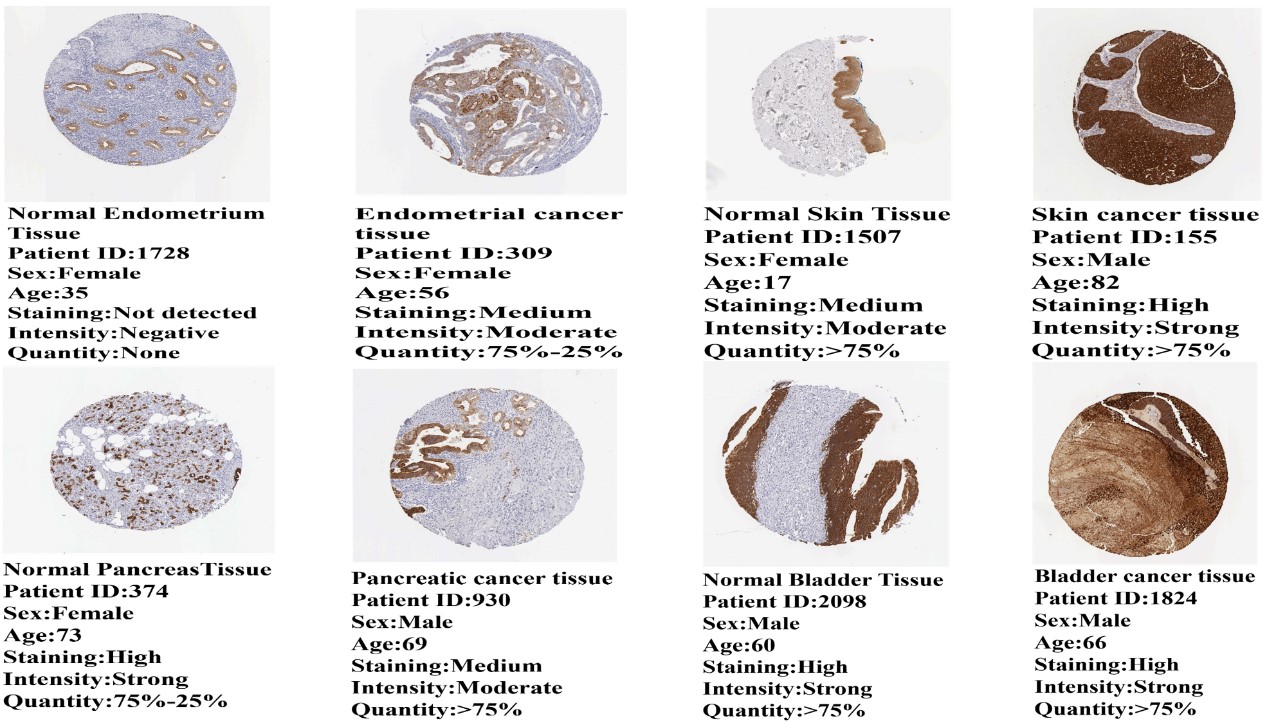


Figure S2c. IHC images of normal and tumor tissues of KRT17 from patients with Endometrial cancer, Skin cancer, Pancreatic cancer and Ovarian cancer. All IHC images and patient information were derived from the HPA.


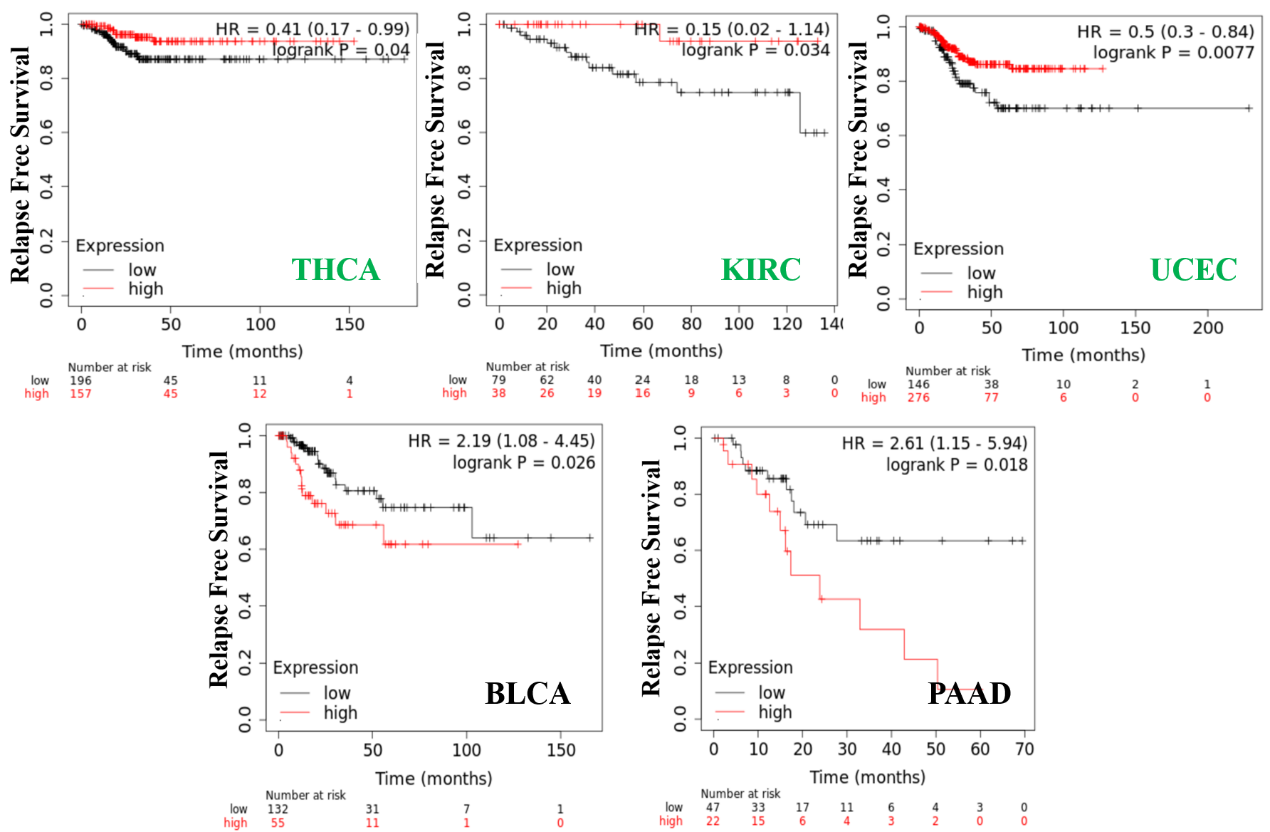


**Figure S3.** KRT17 expression in RFS in THCA, KIRC, UCEC, BLCA and PAAD. We obtained a relationship between KRT17 and survival prognosis of cancer from Kaplan-Meier Plotter. Kaplan-meier curves were all positive. The solid red line represents the high expression of KRT17 in tumor tissues, and the solid black line represents the low expression of KRT17 in tumor tissues.


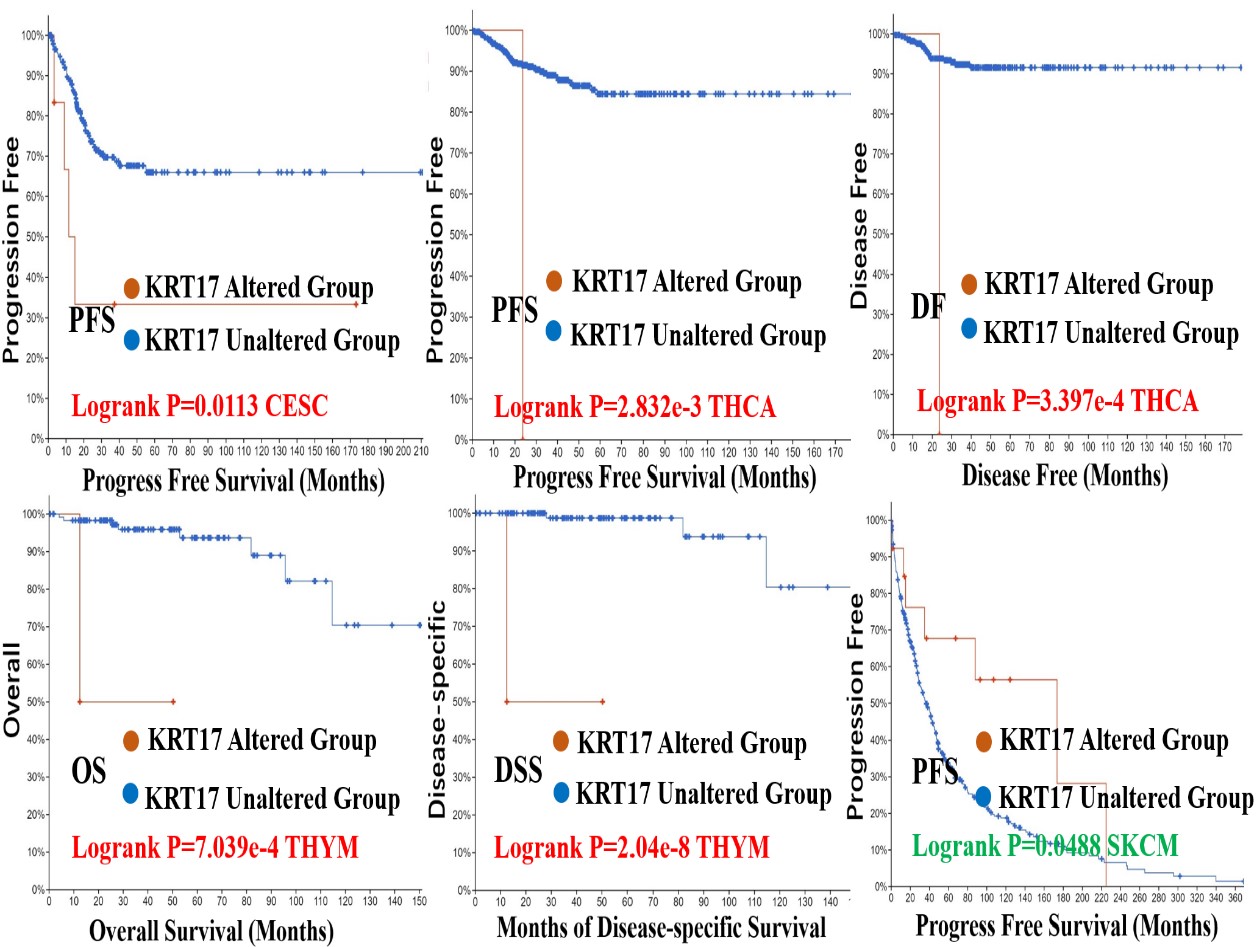


**Figure S4.** Correlation between KRT17 mutation status and PFS of CESC, PFS and DF of THCA, OS and DSS of THYM, and PFS of SKCM. The solid red line represents KRT17 altered group in tumor tissues, and the dash-dotted blue line represents KRT17 unaltered group in tumor tissues. （TCGA dataset）


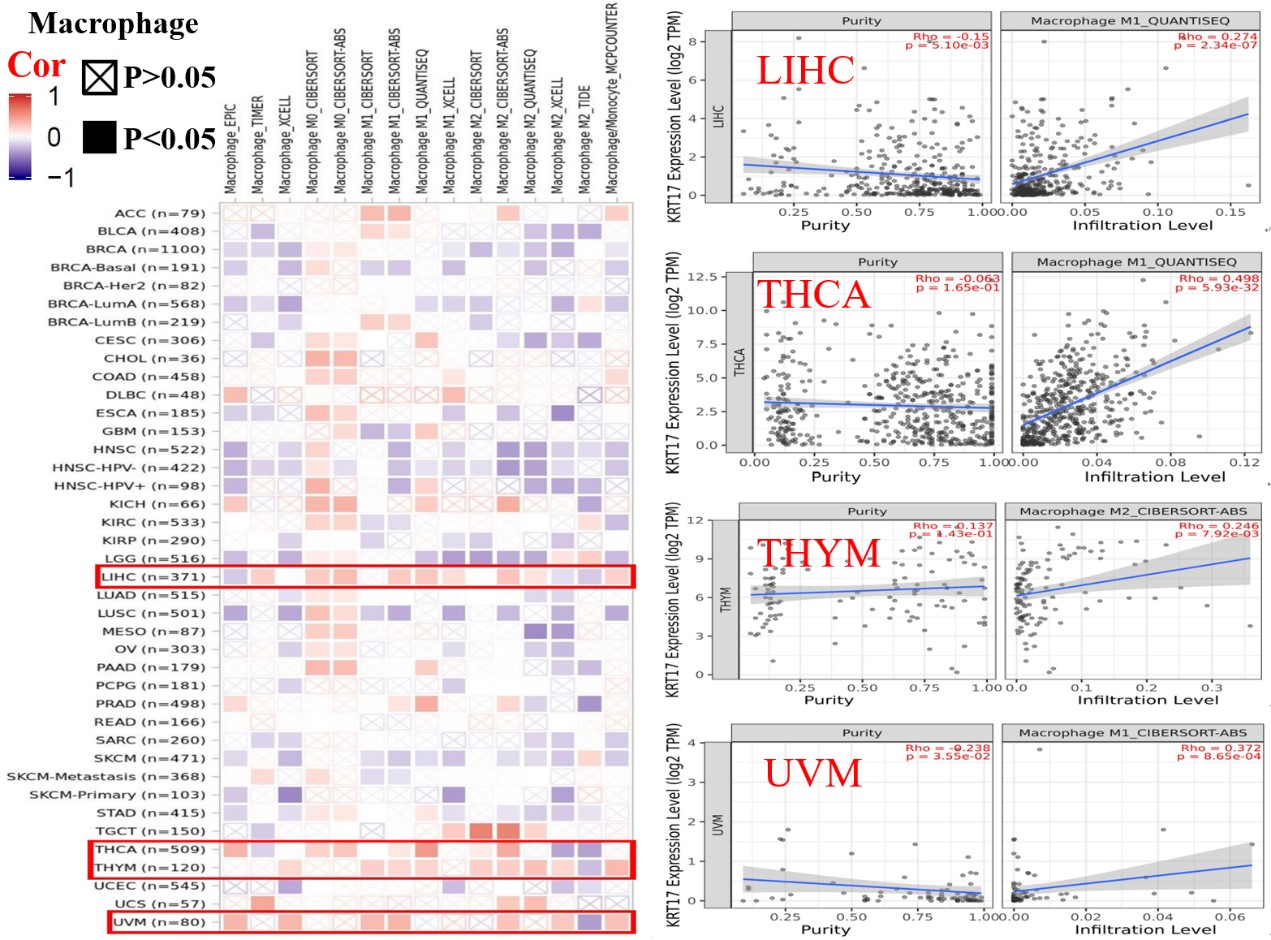


**Figure 5a.** Correlation between KRT17 expression and immune infiltration of Macrophages. In LIHC, THCA, THYM and UVN, KRT17 expression was positively correlated with Macrophages expression.（TCGA dataset）.


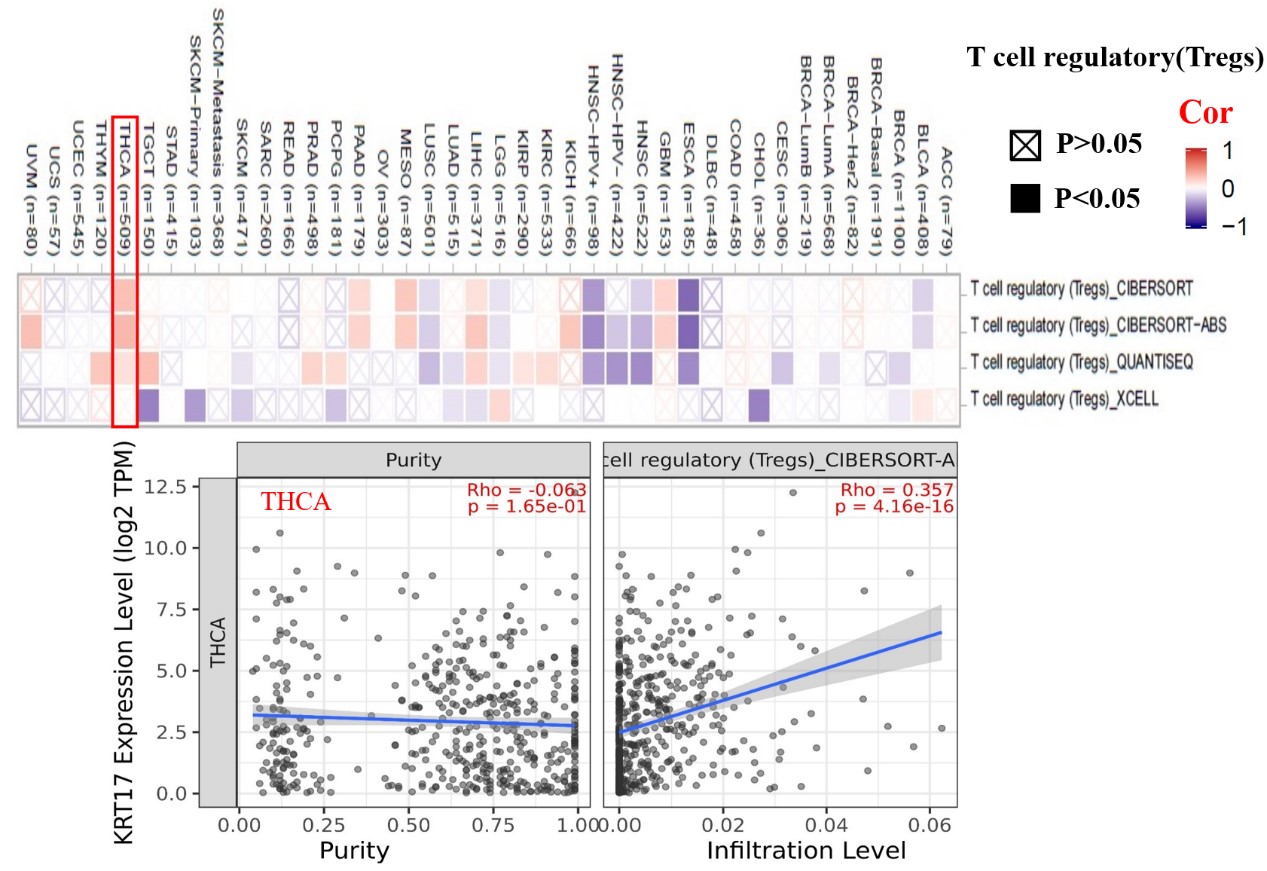


**Figure S5b.** Correlation between KRT17 expression in THCA and immune infiltration of Tregs cells. In THCA, KRT17 expression was positively correlated with Tregs cells.（TCGA dataset）


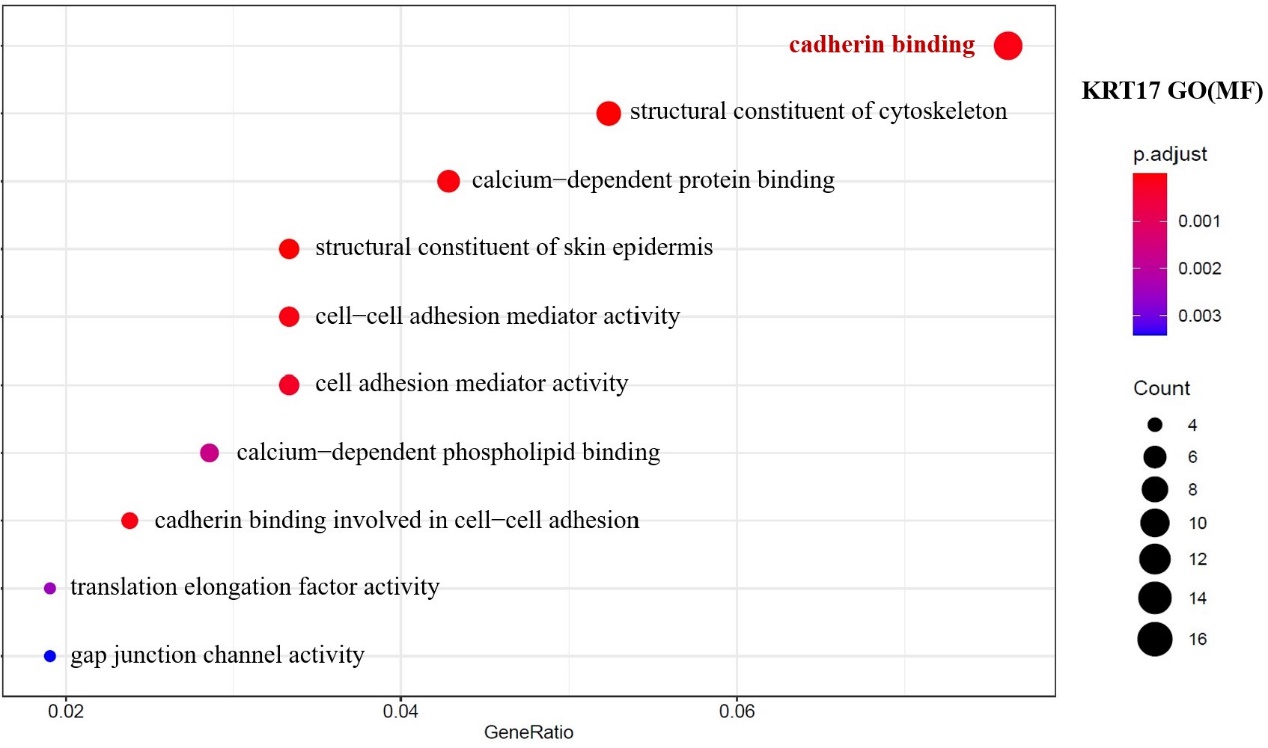
**Figure S6a.** Go (molecular function MF) analysis of KRT17 interaction binding and expression related genes. The most important molecular function is cadherin binding.


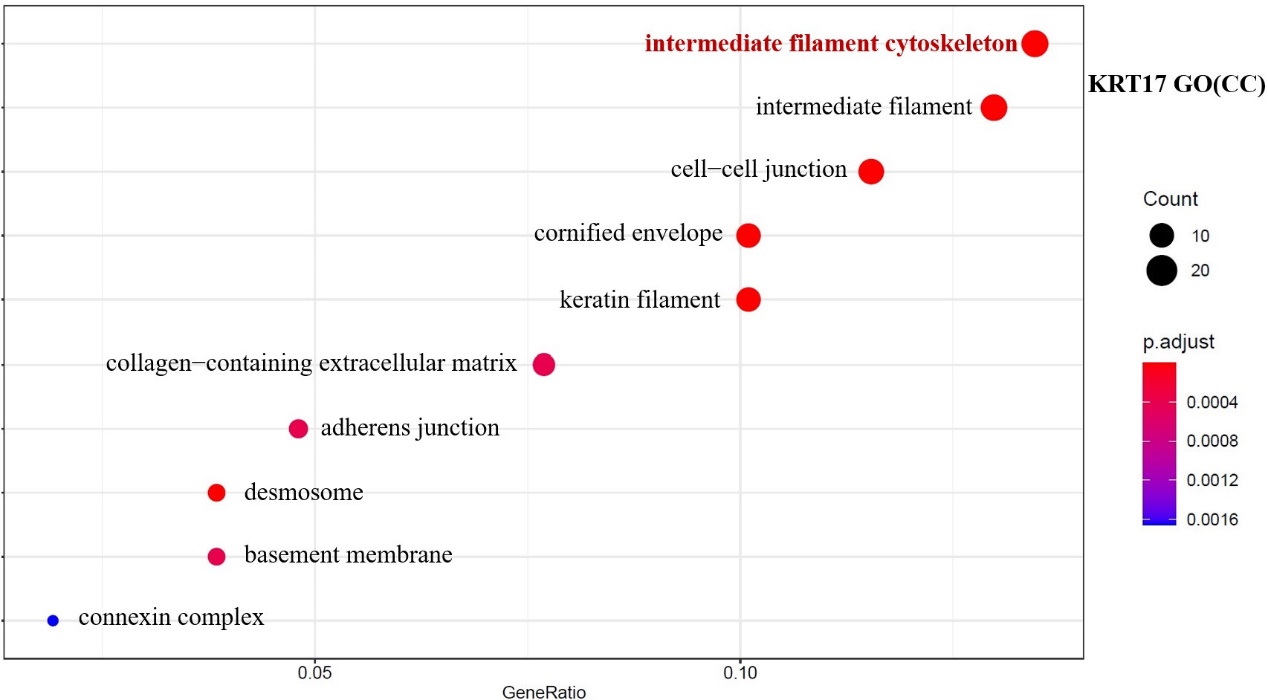


**Figure S6b**. Go (Cellular Component CC) analysis results of KRT17 interaction binding and expression related genes. The most important cellular component is intermediate filament cytoskeleton.


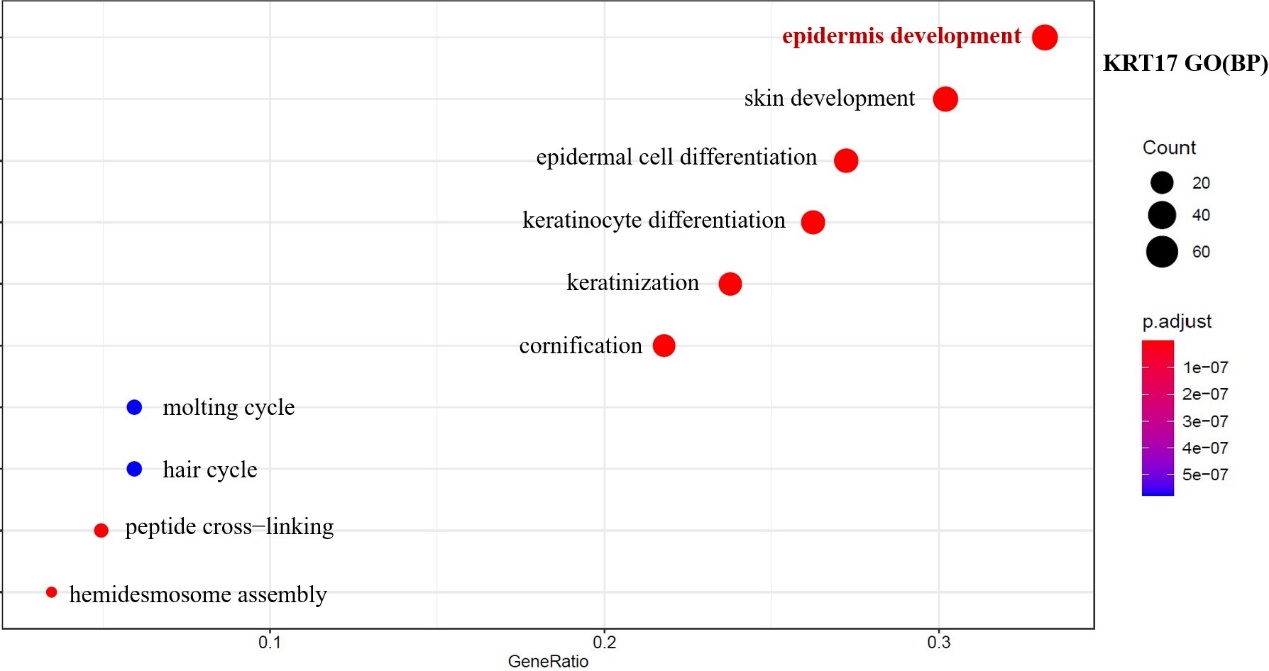


**Figure S6c.** Go (Biological Process BP) analysis results of KRT17 interaction binding and expression related genes. The most important biological process is epidermis development.

**Table 1. IHC intensity of KRT17 in various types of tumors**

| Tumor types | Case number | IHC intensity of KRT17 | | | |
| --- | --- | --- | --- | --- | --- |
|  |  | Strong | Moderate | Weak | Negative |
| Thyroid cancer | 4 | 3 | 0 | 1 | 0 |
| Head and Neck cancer | 4 | 4 | 0 | 0 | 0 |
| Lung cancer | 11 | 6 | 3 | 1 | 1 |
| Liver cancer | 11 | 2 | 2 | 0 | 7 |
| Pancreatic cancer | 12 | 10 | 2 | 0 | 0 |
| Stomach cancer | 11 | 7 | 4 | 0 | 0 |
| Prostate cancer | 12 | 4 | 6 | 0 | 2 |
| Renal cancer | 11 | 0 | 3 | 2 | 6 |
| Bladder cancer | 12 | 12 | 0 | 0 | 0 |
| Ovarian cancer | 10 | 5 | 4 | 1 | 0 |
| Endometrial cancer | 11 | 6 | 5 | 0 | 0 |
| Testis cancer | 11 | 1 | 0 | 1 | 9 |
| Skin cancer | 10 | 10 | 0 | 0 | 0 |
| Glioma | 12 | 0 | 0 | 4 | 8 |
| Lymphoma | 8 | 0 | 0 | 0 | 8 |
| Melanoma | 9 | 1 | 0 | 0 | 8 |

IHC: immunohistochemical
